# Identification of Bacterial Networks and Relationship to Host Responses in Early Periodontitis Population over 24 Months

**DOI:** 10.3390/ijms262210823

**Published:** 2025-11-07

**Authors:** Aaron R. Biesbrock, Sancai Xie, Ping Hu, Cheryl S. Tansky, Xingtao Wei, Hao Ye, Benjamin Circello, Avi Zini, Guy Tobias, Makio Tamura, Mirjana Parlov

**Affiliations:** 1 Global Oral Care R&D, The Procter & Gamble Company, Mason, OH 45040, USA; ye.h@pg.com; 2 Corporate Functions R&D, The Procter & Gamble Company, Mason, OH 45040, USA; xie.s@pg.com (S.X.); hu.p@pg.com (P.H.); wei.x.3@pg.com (X.W.); circello.bt@pg.com (B.C.); tamura.m.6@pg.com (M.T.); parlov.mp@pg.com (M.P.); 3 Global Baby, Feminine, Family Care Life Sciences, The Procter & Gamble Company, Cincinnati, OH 45224, USA; tansky.cs@pg.com; 4 School of Dental Medicine, Hebrew University and Hadassah, Jerusalem 91120, Israel; ddental@savion.huji.ac.il (A.Z.); guy.tobias@mail.huji.ac.il (G.T.)

**Keywords:** stannous fluoride, cetylpyridinium chloride, DNA, periodontal disease, microbiota, proinflammatory cytokines, metalloproteinases, clusters, networks

## Abstract

This research examined the effects of daily application of an oral hygiene regimen on the subgingival microbiome over 24 months. Generally healthy adults (107 enrolled, 87 completed) with early periodontitis used a home-care regimen (stannous fluoride paste, cetylpyridinium chloride rinse, power toothbrush, and floss) or usual care (control). Subgingival plaque samples were analyzed enzymatically for bacterial toxins. TLR ligands were measured using TLR-SEAP and TLR-ATP assays. Proinflammatory cytokines and metalloproteinases were quantified via immunoassays. Subgingival DNA was sequenced using a shotgun approach to assess microbial diversity. Increasing levels of bacteria, toxins, TLR activation, inflammatory cytokines, and MMPs were observed for periodontitis versus gingivitis and gingivitis versus healthy sites. The regimen significantly reduced levels of the critical proinflammatory cytokine IL-1β, as well as MMP-1 and MMP-9, at 24 months. By month 6, TLR ligands within subgingival plaques decreased. The abundance of pathogenic bacteria correlated with levels of virulence factors, proinflammatory cytokines, MMPs, and severity of clinical measures. Two distinct constellations of pathogenic bacteria were identified. Gingival sites were categorized into responders and non-responders per clinical symptoms and biomarkers. The regimen yielded more responder sites (70%) versus the control (47%), *p* = 0.0002914. The regimen reduced pathogenic bacteria, IL-1β, MMP1, and MMP-9, paralleling clinical reductions in periodontal disease.

## 1. Introduction

Periodontal disease is an inflammatory disease of the periodontium that has a microbial etiology. Gingivitis, the earliest form of periodontal disease, is a reversible inflammation of the gingiva characterized by redness, swelling, and bleeding of the gingiva. Periodontitis, a more advanced form of periodontal disease, is irreversible and is characterized by connective tissue destruction of the periodontal ligament and supporting alveolar bone, leading to increased pocket depth and loss of attachment. Periodontitis is one of the leading causes of tooth loss globally. The prevalence of periodontal disease has been reported to range from 20 to 50% globally [1]. Severe periodontitis has been reported to be the 11th most prevalent condition worldwide by the Global Burden of Disease Study [2]. The National Health and Nutrition Examination Survey data from 2009 to 2014 reported that 42.2% of US adults 30 years of age or older had periodontitis, with 7.8% having severe periodontitis and 34.4% having non-severe periodontitis [3]. Periodontal disease treatment targets the microbial etiology of the disease, with scaling and root planing, often supplemented with systemic antibiotic therapy, being the first course of therapy. Periodontal surgery is often indicated to repair osseous pathology and reduce pocket depth, making it easier for the patient to maintain an optimal oral hygiene regimen. Patients who have undergone periodontal therapy enter long-term periodontal maintenance therapy, with a goal of maintaining a healthy microbiome. This approach helps reduce virulence factors and mitigate proinflammatory immune responses, contributing to overall periodontal health.

The microbial etiology of periodontal disease has been described as a subgingival microbial dysbiosis leading to shifts in microbiome virulence that trigger a host inflammatory response. In 1998, Socransky et al. identified microbial complexes in subgingival plaque that were associated with clinical measures of periodontal disease, including pocket depth and bleeding on probing [4]. The red complex, which includes *Porphyromonas gingivalis*, *Tannerella forsythia* (previously known as *Bacteroides forsythus*), and *Treponema denticola*, exhibited a very strong relationship with pocket depth, increasing in both number and prevalence with increasing pocket depth [4]. The orange complex included *Prevotella intermedia*, *Fusobacterium nucleatum*, *Prevotella nigrescens*, *Peptostreptococcus micros*, *Campylobacter rectus*, *Campylobacter showae*, *Campylobacter gracilis*, *Eubacterium nodatum*, and *Streptococcus constellatus* and was closely associated with the red complex bacteria, as well as showing a significant association with increasing pocket depth [4]. In 2021, a subgingival plaque biofilm study of periodontitis patients versus healthy controls reported that *T. forsythia*, *Filifactor alocis*, *Parvimonas micro*, *P. intermedia*, and *T. denticola* were overabundant in periodontitis patients versus healthy controls [5]. Separately, *P. gingivalis*, *T. forsythia*, *F. nucleatum*, *T. denticola*, and *F. alocis* were reported to have increasing abundance in the subgingival plaque of periodontitis sites versus gingivitis sites versus healthy sites [6]. Total abundance of the red complex species was 34% in periodontitis sites, 13% in gingivitis sites, and 4% in healthy sites [6]. Furthermore, bacterial activity assessed by mRNA/16S RNA read abundance demonstrated that the red complex bacteria showed increased activity with disease progression and that *F. alocis* and *Fastibacterium fastidiosum* had even greater transcriptional activity, suggesting a potential role in disease pathogenesis [6]. Ovsepian et al. found that the top 10 overabundant bacterial species in the subgingival plaque of 54 periodontitis versus healthy subjects were *F. alocis*, *T. denticola*, *T. forsythia*, *P. intermedia*, *T. putidum*, *P. multiformis*, *P. gingivalis*, *P. oris*, *C. rectus*, and *D. oralis* [7]. Another study based on salivary sampling of 72 subjects found that the relative abundance of *P. gingivalis*, *T. forsythia*, *F. alocis*, and *P. intermedia* was statistically significantly increased as disease severity increased from healthy to gingivitis to moderate periodontitis to severe periodontitis [8]. In that study, *P. gingivalis*, *T. forsythia*, and *F. alocis* were positively correlated to the sum of full mouth pocket depth with r^2^ = 0.489, 0.342, and 0.495, respectively [8].

A number of the oral bacteria associated with periodontal disease produces virulence factors that interact with the host to initiate and propagate periodontal inflammation. One of the most studied virulence factors is lipopolysaccharide (LPS), also known as endotoxin, which is a structural component of Gram-negative bacterial outer membranes that has a proinflammatory effect on a number of host cells, including gingival keratinocytes, fibroblasts, neutrophils, macrophages and lymphocytes [9]. LPS is a key agonist of Toll-like receptors (TLRs) on host cells that, when bound to the TLR, activates host cell signaling, leading to increased proinflammatory cytokine upregulation, including interleukin-1β (IL-1β) [10]. Putative oral pathogens, such as *P. gingivalis*, *P. pallens*, *T. forsythia*, and *F. nucleatum*, produce LPS that is implicated in bacterial virulence. Beyond stimulating TLR-mediated inflammation, *P. gingivalis* LPS has been demonstrated to degrade gingival epithelial barrier function and induce alveolar bone loss [11,12]. Zaric et al. reported that subgingival endotoxin activity was significantly associated with local probing pocket depth, with a correlation coefficient of 0.63 (*p* < 0.0001) when comparing 33 periodontally healthy subjects to 32 periodontitis subjects [13]. Furthermore, subgingival endotoxin levels had high specificity and high sensitivity to detecting periodontally healthy and diseased sites, with a specificity of 0.91 and a sensitivity of 0.85, implying that subgingival endotoxin activity had a good prognostic value, coupled with the fact that periodontal treatment decreased the activity of endotoxin significantly [13]. Importantly, LPS triggers inflammasome activation, a crucial process in the immune system that leads to the production of IL-1β. IL-1β is a keystone proinflammatory cytokine that coordinates the acute inflammatory response in gingival inflammation. Biesbrock & Yeh reported that in an experimental gingivitis study with 30 subjects, IL-1β concentrations of sampled keratinocytes were positively correlated to the number of clinical bleeding sites with r = 0.90 (*p* < 0.05) using linear regression analysis, which coincided with the development of a subgingival dysbiotic plaque [14].

This paper reports the follow-up analyses of subgingival plaque samples, including the subgingival crevicular fluid, from a 2-year clinical study involving 87 subjects (107 enrolled, 87 finished) with gingivitis/early periodontitis, with the clinical results having been published previously [15]. Baseline demographic and clinical characteristics are shown in Table 1. The study compared an oral health regimen (oscillating–rotating power toothbrush, 0.454% stannous fluoride (SnF_2_) toothpaste, 0.07% cetylpyridinium chloride (CPC) mouthrinse, dental floss and dental prophylaxis every 6 months) to usual care/control (manual toothbrush, 0.245% sodium fluoride (NaF) toothpaste and dental prophylaxis every 6 months). The clinical results for Gingival Bleeding Index (GBI)-bleeding sites demonstrated that the regimen group had a statistically significant (*p* < 0.001) reduction of 33%, 28%, 38%, and 39% at 6, 12, 18, and 24 months, respectively, versus the usual care treatment group [15]. Similarly, the clinical results for probing pocket depth (PPD) adjusted means demonstrated that the regimen group had a statistically significant (*p* < 0.001) reduction of 12%, 6%, 11%, and 13% at 6, 12, 18, and 24 months, respectively, versus the usual care treatment group [15]. Lastly, the median number of ≥2 mm PPD loss events demonstrated that the regimen group had a statistically significant (*p* < 0.005) reduction of 73%, 56%, 77%, and 74% at 6, 12, 18, and 24 months versus the usual care treatment group [15]. The subgingival plaque samples in this paper were collected from 9 sites from each subject, with 3 healthy sites (≤3 mm PD with no bleeding on probing (BOP)), 3 gingivitis sites (≤3 mm PD with BOP), and 3 periodontitis sites (≥5 mm PD with BOP). These subgingival plaque samples were analyzed for microbial sequencing, LPS, TLR–adenosine triphosphate (ATP), TLR-secreted embryonic alkaline phosphatase (SEAP), and proinflammatory cytokines. Specifically, the primary objective of the analyses was to establish the differences in bacterial composition across healthy, gingivitis and periodontitis sites at baseline, with a secondary objective of examining treatment effects of regimen versus control at the end of the study. Beyond these objectives, the correlation between the bacteria of subgingival plaque with clinical symptoms and biomarkers was explored, including the identification of bacterial networks that were associated with periodontal disease in this study.

## 2. Results

### 2.1. Microbial DNA Yields and Diversity in Subgingival Plaques: Baseline Disease Severity and Treatment Effects After 24 Months

**Figure 1:**
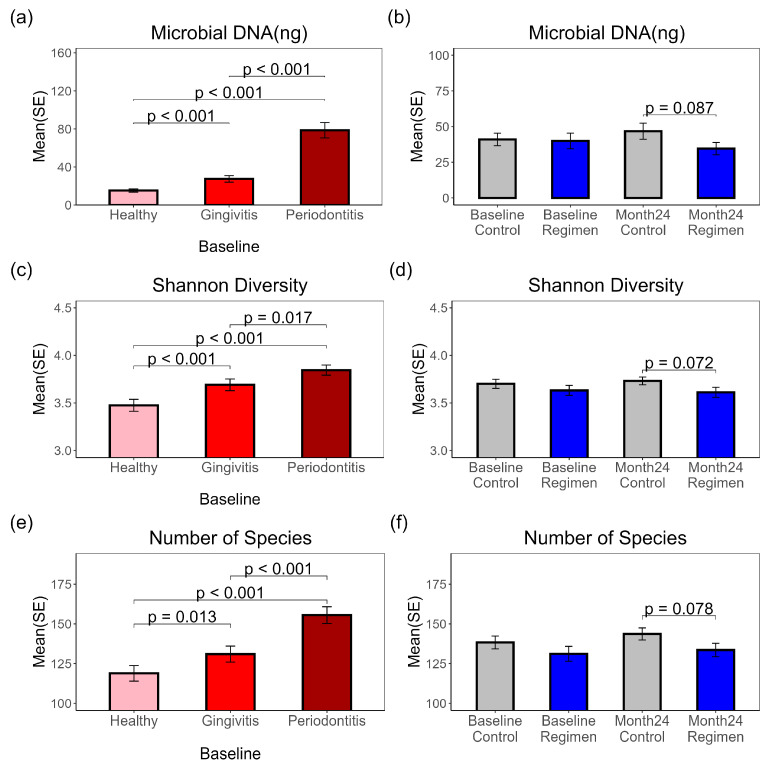
Total Microbial DNA, Shannon diversity and number of microbial species in subgingival plaques. Pairwise *t*-test results are shown for comparisons with *p* ≤ 0.1. (**a**) Microbial DNA amounts at baseline from healthy, gingivitis and periodontitis sites. (**b**) Microbial DNA amounts before and after 24-month treatment. (**c**) Shannon Diversity Index at baseline from healthy, gingivitis and periodontitis sites. (**d**) Shannon Diversity Index before and after 24-month treatment. (**e**) Observed microbial species number at baseline from healthy, gingivitis and periodontitis sites. (**f**) Observed microbial species before and after 24-month treatment.

Total DNA was purified from subgingival plaques collected from three sites (each) labeled as healthy, gingivitis, or periodontitis before and after 24 months of treatment for metagenomic sequencing and microbiome analysis. Sequences mapped to the human genome were separated from those mapped to microbial genomes. The corresponding total microbial DNA was calculated by multiplying the total purified DNA by the ratio of mapped microbial sequences for all species to the total sequence count for each sample. Significant differences in microbial DNA were observed at baseline (Figure 1a) among the periodontitis (78.70 ng), gingivitis (27.46 ng), and healthy (15.31 ng) sites, suggesting a potential link between disease severity and microbial mass. After the 24-month treatment, the regimen group exhibited lower microbial DNA levels in their plaques compared to the control group (*p* = 0.087) (Figure 1b). Both Shannon diversity (Figure 1c) and observed microbial species (Figure 1e) significantly differed at baseline, with the highest levels in periodontitis samples, followed by gingivitis, and the lowest in healthy samples (Table S1, Figure S1). The 24-month regimen treatment also reduced Shannon diversity (Figure 1d) (*p* = 0.072) and observed species (Figure 1f) (*p* = 0.078) compared to the control treatment.

### 2.2. Evaluation of Microbial Abundance in Subgingival Plaques and Its Correlation with Disease Severity

**Figure 2:**
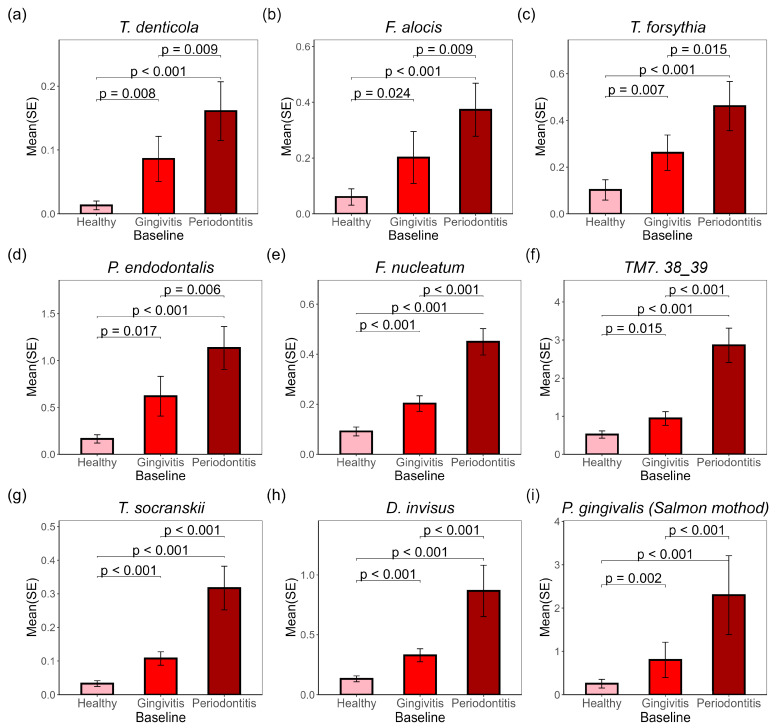
Selected bacteria species associated with gingivitis biomarkers were enriched in gingivitis and periodontitis sites in subgingival plaques at baseline. *p*-values for pairwise *t*-tests are marked on the graph. The representative microbial amounts (ng) at baseline from healthy, gingivitis, and periodontitis sites include: (**a**) *T. denticola*; (**b**) *F. alocis*; (**c**) *T. forsythia*; (**d**) *P. endodontalis*; (**e**) *F. nucleatum*; (**f**) *TM7 38_39*; (**g**) *T. socranskii*; (**h**) *D. invisus*; (**i**) *P. gingivalis*. Figures (**a**–**h**) were generated using MetaPhlAn data, while Figure (**i**) was generated using Salmon data.

The relative abundance of detected microbial species was calculated and multiplied by the microbial DNA amount for each sample to determine the absolute biomass of each species. Significant differences were observed at baseline among the three sample types, as illustrated in Figure 2a–i. For each bacterial species in Figure 2, periodontitis sites exhibited the highest DNA amount, inked to periodontal diseases included *T. denticola* (Figure 2a), *F. alocis* (Fifollowed by gingivitis sites, with the lowest levels found in healthy sites. Notable oral pathogens lgure 2b), *T. forsythia* (Figure 2c), *P. endodontalis* (Figure 2d), *F. nucleatum* (Figure 2e) and *P. gingivalis* (Figure 2i). Other species, such as *TM7 38_39* (Figure 2f), *T. socranskii* (Figure 2g), and *D. invisus* (Figure 2h), also showed significant changes despite being less associated with periodontal diseases.

Metaphlan4 was initially used for microbial species detection, which relies on single-copy or low-copy gene matching; this approach can sometimes lead to under-detection or misclassification of key species. For instance, only 13.14% of our samples detected *P. gingivalis*, compared to 61.37% for *P. endodontalis.* To address this, a customized oral genome library was constructed with genes from 83 common oral bacterial strains, and the genomic sequences were mapped to this library to calculate relative percentages and absolute amounts (Salmon method). Three *P. gingivalis* strains were included in this library—*W83*, *ATCC33277*, and *TDC60.* These three strains are commonly used in periodontitis research to identify unique genes, proteins, and biological functions, including specific virulence factors and metabolic pathways.

Using this targeted identification method, *P. gingivalis ATCC33277* and *P. endodontalis* were detected in all samples, while *P. gingivalis W83* and *TDC60* were found in over 80% of samples. The DNA levels of all three *P. gingivalis* strains varied significantly among healthy, gingivitis, and periodontitis sites, with the highest levels in periodontitis sites and the lowest in healthy sites. *P. gingivalis ATCC 33277* is considered to have more virulent genes in its genome compared to the other two strains; it shows higher abundance in the baseline samples than the other two strains (Figure S2).

### 2.3. Endotoxins, Gingipains and Peptidylarginine Deiminases (PADs)

**Figure 3:**
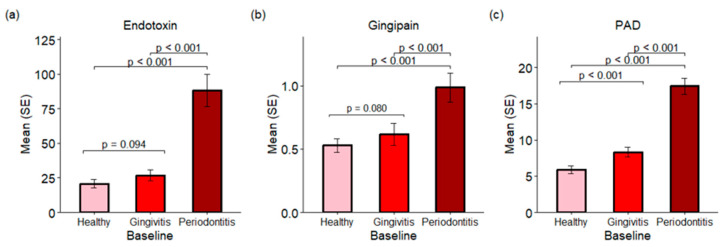
Endotoxins, gingipains and PADs in subgingival plaques from baseline samples. (**a**) Endotoxins are presented as the mean and standard error (SE) of endotoxin units. (**b**) The activities of gingipains are expressed in RFUs. (**c**) The activities of PADs are also expressed in RFUs.

The investigation into subgingival plaques revealed the presence of important virulence factors, including endotoxins, gingipains, and PADs. The cohort comprised participants with at least three sites (each) representing healthy, gingivitis, and periodontitis conditions. Endotoxin and gingipain activities were significantly elevated in periodontitis sites compared to both gingivitis and healthy sites at baseline (Figure 3a,b) with a *p*-value < 0.001. The average endotoxin levels were 20.77, 26.75, and 88.46 endotoxin units for healthy, gingivitis, and periodontitis sites, respectively. Similarly, the average gingipain levels were 0.65, 0.74, and 1.38 relative fluorescence units (RFUs) for healthy, gingivitis, and periodontitis sites, respectively. In the PAD assay, a substrate that can be converted to ammonia by both human and microbial PADs was employed, indicating total PAD activity. PAD activities were found to be higher in gingivitis sites than in healthy sites (*p* < 0.001), with periodontitis sites exhibiting the highest levels of PAD activity (*p* < 0.001 compared with either healthy or gingivitis sites). A strong correlation was identified between PAD activity and the presence of *P. gingivalis* in the subgingival plaque, with a correlation coefficient of 0.68 (*p* < 0.001) (see Section 2.8). The results suggest that PAD activity in subgingival plaques primarily originated from *P. gingivalis*.

### 2.4. Pathogen-Associated Molecular Patterns (PAMPs) in Subgingival Plaques

**Figure 4:**
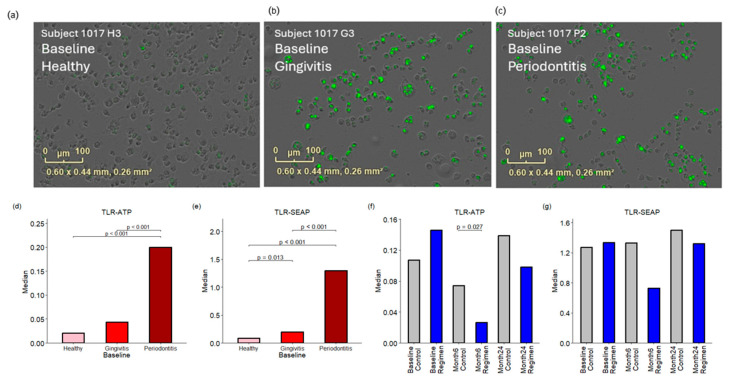
Responses of TLR-ATP biosensor and TLR-SEAP to PAMPs in the subgingival plaques. Panels (**a**–**c**) present images of TLR-ATP biosensor cells responding to treatment with subgingival plaques collected from a single subject, which represents the median response for healthy, gingivitis, and periodontitis sites, respectively, at baseline. The green spots represent green fluorescence of TLR-ATP biosensor. Panels (**d**,**e**) compare the baseline responses observed in both the TLR-ATP biosensor and TLR-SEAP assays. Panels (**f**,**g**) illustrate the effects of the treatment regimen at six months and 24 months, respectively.

Pathogen-associated molecular patterns (PAMPs) in subgingival plaques were quantified using two TLR reporter assays. The TLR-ATP biosensor assay evaluates the metabolic activity of reporter cells in response to PAMPs, leading to increased intracellular ATP levels. This rise in ATP triggers a fusion GFP protein to emit green fluorescence, which serves as an indicator of enhanced cellular metabolism. See Figure 4. The findings indicated *P. gingivalis* lipopolysaccharide (LPS) equivalents in μg/mL, with median values of 0.02, 0.04, and 0.20 for healthy, gingivitis, and periodontitis sites, respectively, at baseline. A significant increase in green fluorescent protein (GFP) fluorescence was detected in plaques from periodontitis patients, with a *p*-value of <0.001 compared to both healthy and gingivitis sites at baseline. The test regimen showed a significant reduction in fluorescence levels at the six-month mark (*p* < 0.027) compared to the control group, with medians of 0.07 for the control group and 0.03 for the regimen group.

Additionally, the TLR-SEAP assay, a standard gene reporter assay, was utilized to further investigate the response to bacterial PAMPs. The results were quantified as absorbance at 625 nm, with baseline median values of 0.08, 0.20, and 1.30 for healthy, gingivitis, and periodontitis sites, respectively. Plaques from periodontitis exhibited the strongest responses in the TLR-SEAP assay, with *p*-values < 0.001 when compared to both healthy and gingivitis sites. Furthermore, plaques from gingivitis showed significantly elevated TLR-SEAP responses (*p* = 0.013) compared to healthy sites.

### 2.5. Proinflammatory Cytokine Concentrations in Gingival Crevicular Fluid (GCF)

Proinflammatory cytokine concentrations in GCF were assessed using the V-PLEX Proinflammatory Panel 1 Human Kit, which measures various cytokines, including IFN-γ, IL-1β, IL-2, IL-4, IL-6, IL-8, IL-10, IL-12p70, IL-13, and TNF-α. Results are expressed in picograms per milliliter (pg/mL). Among these, IL-1β is a key proinflammatory cytokine that plays a vital role in the immune response to bacterial infections.

**Figure 5:**
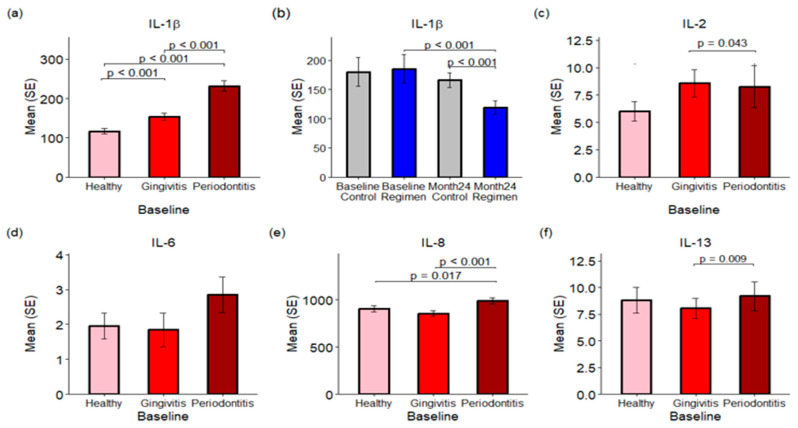
Proinflammatory Cytokine Concentrations in Gingival Crevicular Fluid (GCF). Panels (**a**,**c**–**f**) illustrate the concentrations of various proinflammatory cytokines in GCF across healthy, gingivitis, and periodontitis sites at baseline. Panel (**b**) specifically compares IL-1β levels at baseline and after 24 months between the control and test regimen treatment groups.

The concentrations of IL-1β in GCF were found to be 116.55 pg/mL for healthy sites, 153.06 pg/mL for gingivitis sites, and 215.69 pg/mL for periodontitis sites (see Figure 5a) at baseline. The level of IL-1β in gingivitis sites was significantly higher than that in healthy sites (*p* < 0.001), but lower than in periodontitis sites (*p* < 0.001). The concentrations of TNF-α were found to be 3.75 (pg/mL) for healthy sites, 3.43 (pg/mL) for gingivitis sites, and 4.47 (pg/mL) for periodontitis sites at baseline. The level of TNF-α in gingivitis sites was significantly lower than in periodontitis sites (*p* = 0.038), but there was no statistically significant change when comparing healthy sites to gingivitis sites and periodontitis sites.

Treatment effects were assessed by combining results from healthy sites along with those affected by gingivitis and periodontitis. Treatment with the regimen led to a significant reduction in IL-1β levels in GCF over a 24-month period when compared to the control group (*p* < 0.001), with concentrations of 166.34 pg/mL for the control group and 115.90 pg/mL for the test regimen (see Figure 5b).

In addition to IL-1β, levels of IL-2, IL-8, and IL-13 were significantly elevated in the GCF of periodontitis sites compared to healthy and gingivitis sites (*p* < 0.05; see Figure 5c–f). Conversely, IFN-γ, IL-4, IL-6, IL-10, and IL-12p70 exhibited no statistically significant changes across healthy, gingivitis, and periodontitis sites.

### 2.6. Matrix Metalloproteinases (MMPs) in GCF

**Figure 6:**
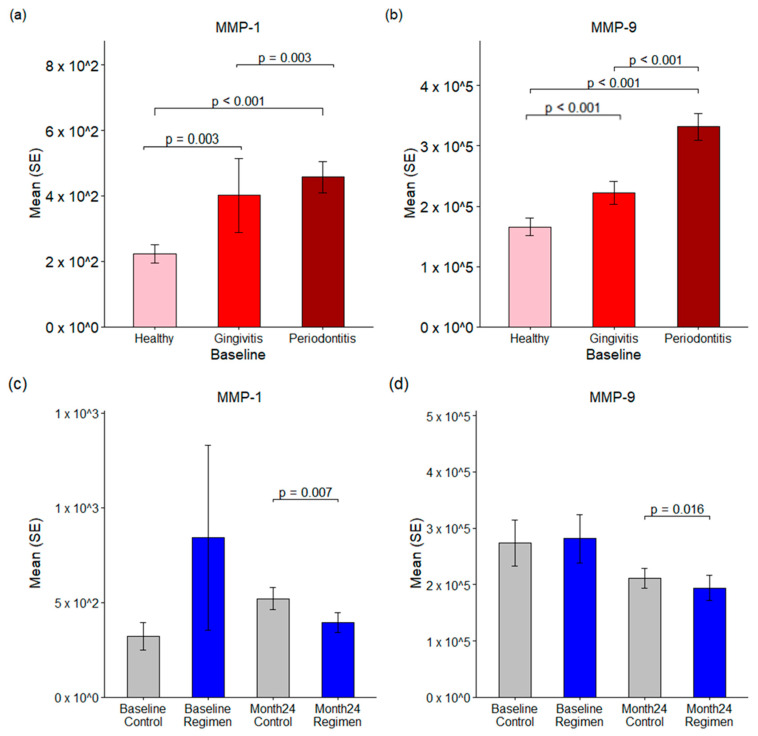
Matrix metalloproteases in GCF. Panels (**a**,**b**) illustrate the baseline levels of MMP-1 and MMP-9 in healthy, gingivitis, and periodontitis sites. Panels (**c**,**d**) compared the changes in MMP-1 and MMP-9 between the control and test regimen groups at both baseline and 24 months.

MMPs are a crucial group of enzymes responsible for the degradation of extracellular matrix components. Elevated levels of MMP1 and MMP9 are frequently associated with pathological conditions. In gingival crevicular fluid (GCF), the concentrations of MMP1 were measured as follows: 223.26 pg/mL in healthy sites, 401.77 pg/mL in gingivitis sites, and 457.02 pg/mL in periodontitis sites (Figure 6a). Statistical analysis indicates that MMP1 levels were significantly higher in gingivitis sites compared to healthy sites (*p* = 0.003) and peaked in periodontitis sites, with a *p*-value of 0.003 when compared to gingivitis.

Moreover, a test regimen resulted in a significant reduction in MMP1 levels after 24 months compared to the control group (*p* < 0.007), with mean concentrations of 519.71 pg/mL in the control group versus 395.35 pg/mL in the test regimen group (Figure 6c).

Similarly, MMP9 levels were found to be abundant in GCF, with baseline concentrations measured at 165.79 ng/mL in healthy sites, 222.01 ng/mL in gingivitis sites, and 331.56 ng/mL in periodontitis sites (Figure 6b). MMP9 levels were significantly elevated in both gingivitis and periodontitis compared to healthy sites, with a *p*-value of ≤0.001. Additionally, the test regimen led to a notable reduction in MMP9 levels after 24 months when compared to the control group (*p* = 0.016), with mean concentrations of 211.0 ng/mL in the control group and 193.8 ng/mL in the test regimen group (Figure 6d). These results underscore the therapeutic effects of the test regimen in reducing MMP levels associated with inflammatory conditions.

### 2.7. Correlation of Bacterial Abundance with Clinical Symptoms, Proinflammatory Cytokines, MMPs, and Virulence Factors

**Figure 7:**
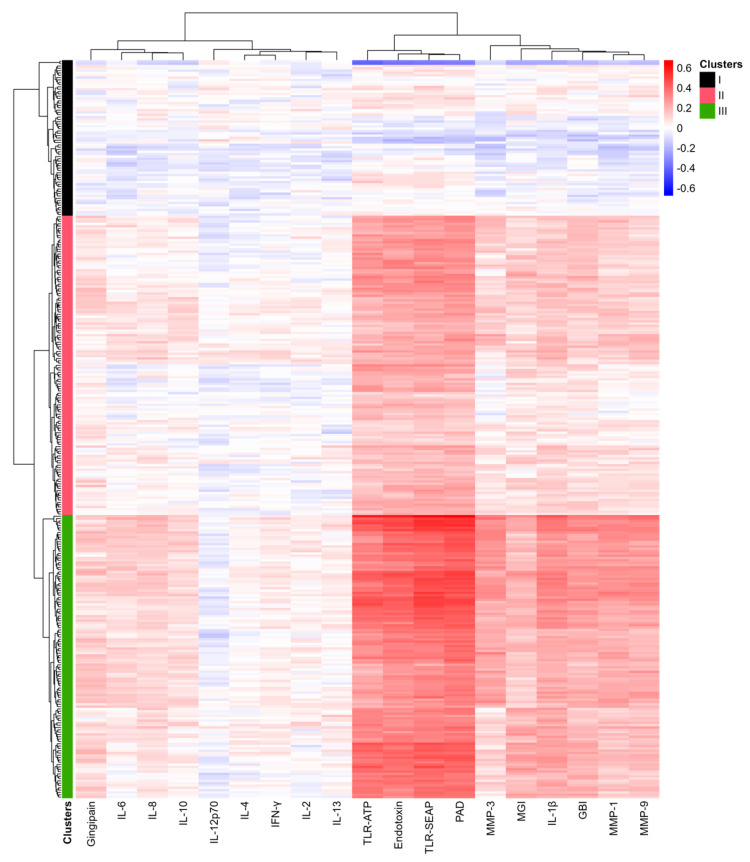
Heat map of correlations between bacterial abundance and clinical measurements, proinflammatory cytokines, MMPs, and virulence factors. Blue indicates negative correlations, while red signifies positive correlations. The magnitude of the correlations is noted in the scale on the upper right. The intensity of the colors reflects the magnitude of the correlation coefficients, with more vibrant colors representing stronger correlations.

To identify the bacteria most strongly correlated with gingivitis and periodontitis, correlation coefficients were calculated for each bacterium detected in this study, leading to the creation of a heatmap (Figure 7). A total of 513 bacterial species were identified in subgingival plaques at baseline from sites categorized as healthy, gingivitis, and periodontitis (see Supplemental Table S1 and Figure S2). Among these, 318 bacterial species that were present in at least 10% of subgingival samples were used to generate the heatmap.

The bacteria were organized into three clusters based on dendrogram analysis (Clusters I to III). In the heatmap, red indicates high positive correlation coefficients, while blue signifies negative correlations. Notably, TLR-SEAP, TLR-ATP, endotoxin levels, and PAD measurements showed very strong correlations with the bacteria in Cluster III. In contrast, IL-1β, MMP-1, MMP-3, MMP-9, GBI, and MGI exhibited more modest correlation coefficients. Additionally, gingipains, IL-8, IL-6, and IL-10 demonstrated weak but positive correlations with the bacteria in Cluster III.

Cluster III, consisting of 122 bacterial species, primarily included all previously identified periodontal pathogens, such as *P. gingivalis*, *P. endodontalis*, *T. denticola*, *T. forsythia*, *F. nucleatum*, and *F. alocis.* Conversely, Cluster I included 67 bacterial species identified as commensals, primarily from the genera *Streptococcus*, *Corynebacterium*, *Rothia*, and *Actinomyces*.

Bacteria in Cluster II, consisting of 129 species, displayed modest but noticeable correlations with TLR-SEAP, TLR-ATP, PAD, endotoxins, IL-1β, MMP-1, MMP-3, MMP-9, GBI, and MGI. These findings suggest that the subgingival plaque community undergoes significant changes as a whole in response to inflammatory and bleeding conditions.

### 2.8. Pathogenic Bacteria Form Networks in Subgingival Plaques

**Figure 8:**
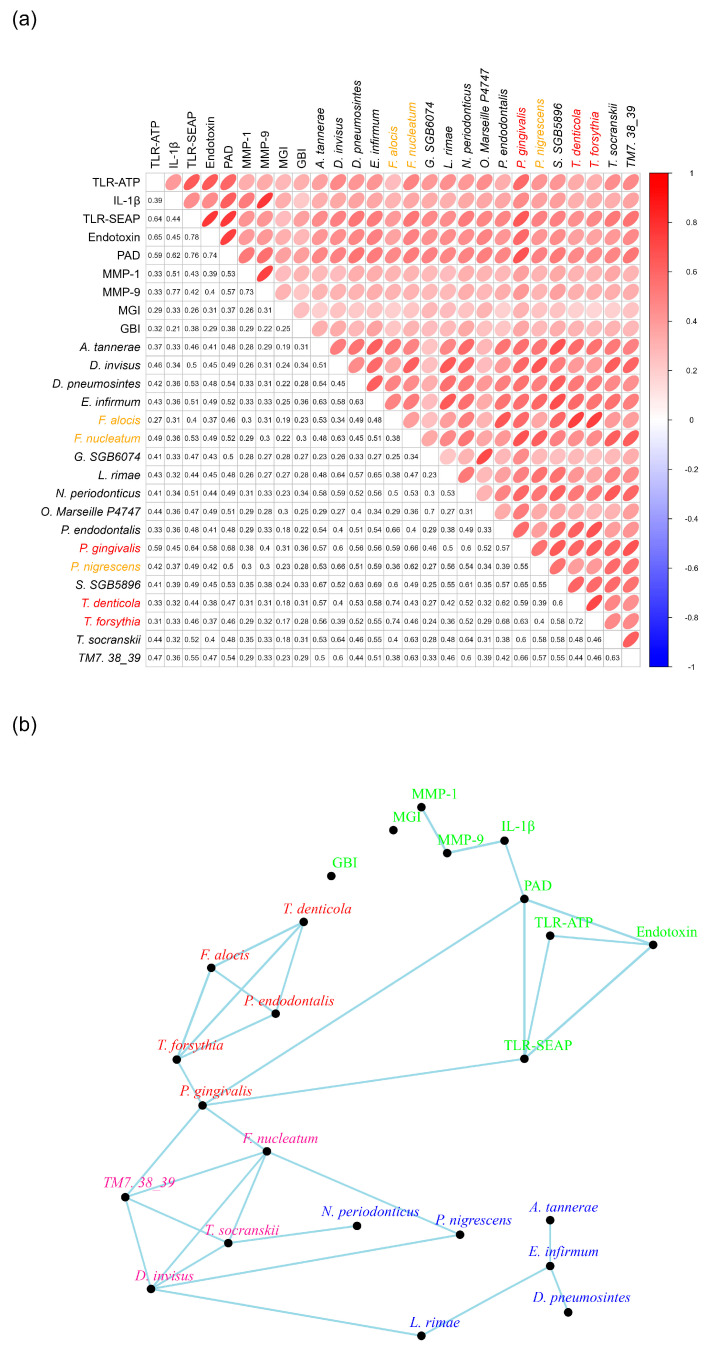
Bacterial Networks in Subgingival Plaques. Panel (**a**) presents the correlation coefficients of the identified bacteria in relation to the nine other measurements. Bacteria labeled in red are classified as members of the red complex, while those labeled in orange are part of the orange complex (reference). Panel (**b**) illustrates the networks among the bacteria and the nine other measurements, highlighting their interconnections.

Nine factors, including IL-1β, endotoxins, TLR-SEAP, TLR-ATP, PAD, MMP-1, MMP-9, GBI, and MGI, exhibited strong and modest correlations with the 122 bacteria in Cluster III. To assess how pathogenic bacterial species were associated with one another, bacterial species were selected based on a *p*-value of <0.05 for each of the nine factors, along with strong correlation coefficients (Figure 8a). To streamline the analysis, only those bacteria with specific species names were included in the subsequent network construction (Figure 8b).

Connections between the bacteria and the nine factors were established when their correlation coefficients were 0.6 or greater. This analysis revealed two distinct bacterial constellations. The first constellation, labeled in red (Figure 8b), *included P. endodontalis*, *T. forsythia*, *T. denticola*, and *F. alocis*, with each species exhibiting correlation coefficients of 0.6 or higher with the other three (Figure 8b).

The second constellation, marked in pink, comprised *F. nucleatum*, *TM7 38-39*, *D. invisus*, and *T. socransii*. For the purpose of network plotting, *TM7 38-39* is abbreviated from *Candidatus nanosynsacchari sp. TM7_ANC_38_39_G1_1*. Each species in this constellation also demonstrated correlation coefficients of 0.6 or greater with the other three bacterial species (Figure 8b).

Interestingly, *P. gingivalis* was absent from both the red and pink constellations. However, it was associated with *T. forsythia* in the red constellation and with both *F. nucleatum* and *TM7 38-39* in the pink constellation. Notably, *P. gingivalis* exhibited strong correlation coefficients with both PAD and TLR-SEAP measurements, specifically 0.68 and 0.64, respectively (Figure 8b). Furthermore, it is important to highlight that *P. gingivalis* produces a PAD enzyme known as *Porphyromonas* PAD.

To enhance the networks among bacteria in the subgingival plaques, the eight bacteria identified in the two constellations were matched with all other bacteria exhibiting correlation coefficients of 0.5 or greater and *p*-values of less than 0.05 (Figure S3). Some of these bacteria demonstrated strong correlations with certain proinflammatory measurements, but not all nine factors. All identified bacteria were categorized within Cluster III.

These bacteria were then utilized to construct networks based on a correlation coefficient threshold of 0.6 (Figure S4). Notably, *C. rectus* and *F. fastidiosum* established connections with *T. forsythia*, *T. denticola*, and *F. alocis* in the red constellation. Meanwhile, *P. maculosa* and *A. dentalis* were linked to *TM7 38-39*, *D. invisus*, and *T. socranskii* in the pink constellation.

### 2.9. Bacterial Networks Independent of Gingivitis and Periodontitis Conditions

**Figure 9:**
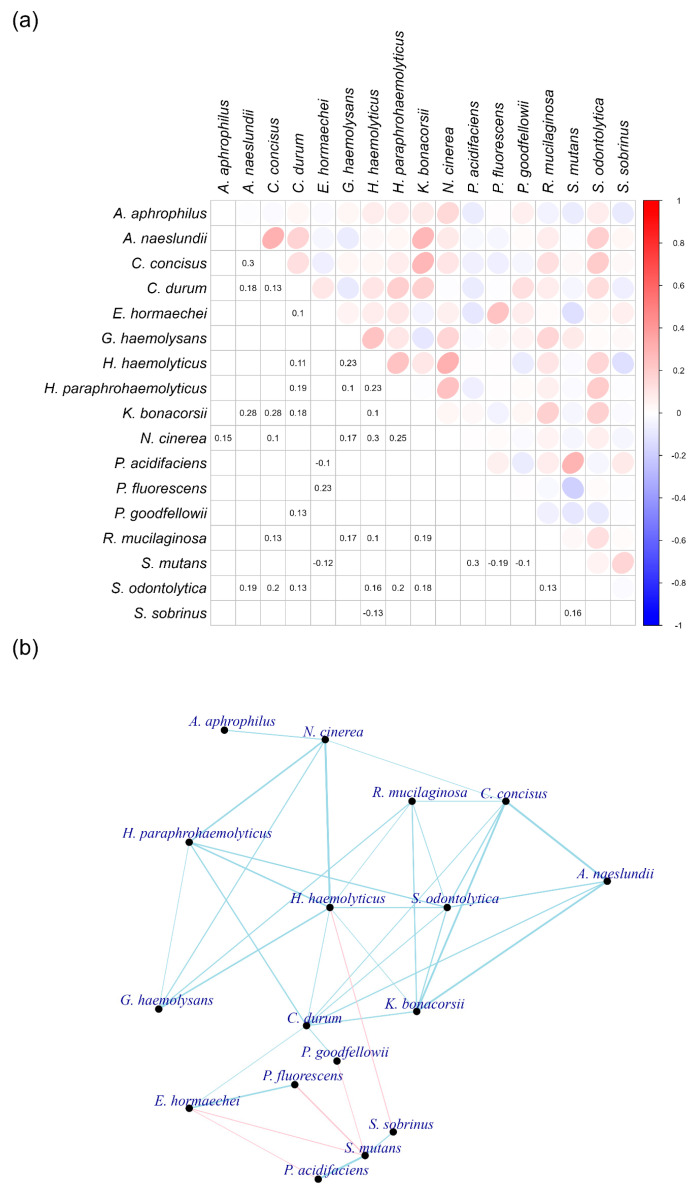
Bacteria that are not correlated with proinflammatory cytokines, bacterial virulence, and clinical symptoms of gingivitis and periodontitis in subgingival plaques. Panel (**a**) displays the correlation coefficients between individual bacterial species. Panel (**b**) illustrates the networks among these bacteria. The thickness of the lines indicates the strength of the correlation coefficients, with blue representing positive correlations and red indicating negative correlations.

Certain bacteria in subgingival plaques appear to be minimally affected by the factors associated with gingivitis and periodontitis, despite most bacteria exhibiting correlations with IL-1β, endotoxins, TLR-SEAP, TLR-ATP, PAD, MMP-1, MMP-9, GBI, and MGI (Figure 7). To identify bacteria that are not correlated with these nine measurements, we selected those for which at least six of the nine factors (IL-1β, endotoxins, TLR-SEAP, TLR-ATP, PAD, MMP-1, MMP-9, GBI, and MGI) had *p*-values greater than 0.05 (Figure 9a).

The selected bacteria did not demonstrate high correlation coefficients to each other, with connections established only for those exceeding 0.1 (Figure 9b). Notably, a bacterium from the *Rothia* genus, *R. mucilaginosa*, exhibited connections with various other bacteria in this constellation. Additionally, two bacteria from the *Haemophilus* genus, *H. haemolyticus* and *H. paraphrohaemolyticus*, displayed multiple connections. It is also noteworthy that *S. mutans* was connected to *S. sobrinus* and *P. acidifaciens*. This group of bacteria showed negative correlations with other species in the constellation.

Over half of the bacteria in this group did not show significant changes in abundance across healthy, gingivitis, and periodontitis states. However, *E. hormaechei* and *N. cinerea* decreased in abundance during periodontitis, while *K. bonacorsii*, *N. cinerea*, and *R. mucilaginosa* increased (Figure S5). The significance of this constellation remains unclear, as its overall abundance was considerably lower than that observed in the red and pink constellations.

Gingivitis and periodontitis environments significantly influenced bacterial abundance in healthy, gingivitis, and periodontitis sites (Figure 2 and Figure S2). This raises the question: Is there a strong association between bacteria that are not correlated with proinflammatory cytokines, bacterial virulence, and clinical symptoms of gingivitis and periodontitis, and those that exhibit strong correlations in subgingival plaques?

To investigate this question, correlations were analyzed among the bacteria identified in both the non-correlated and correlated constellations (Figure S6) and constructed networks (Figure S7). The bacteria in the red and pink constellations of the correlated group displayed strong connections, while those in the non-correlated and correlated groups showed weak interactions. The connections between bacteria correlated and not correlated with gingivitis and periodontitis measurements were primarily negative.

### 2.10. Separation of Responder and Non-Responder Gingival Sites Based on Proinflammatory Cytokine, MMP, Virulence Factor Responses, GBI, and MGI in Gingivitis and Periodontitis

**Figure 10:**
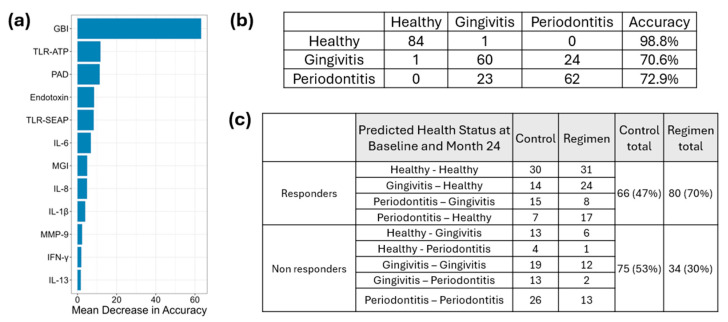
Classification of Responders and Non-Responders Based on Proinflammatory Cytokines, MMPs, Virulence Factor Responses, and Severity of Gingivitis and Periodontitis Using Random Forest Modeling Panel (**a**) illustrates the mean decrease in accuracy. Panel (**b**) presents the prediction accuracy based on the baseline results derived from the developed model. Panel (**c**) displays the predicted outcomes at month 24, informed by proinflammatory cytokines, MMPs, virulence factor responses, and the severity of gingivitis and periodontitis.

Clinical measurements (GBI and MGI), proinflammatory cytokines, MMPs, and microbial virulence factors were analyzed using a Random Forest algorithm, leading to the development of a predictive model (Figure 10a) based on baseline results. This model demonstrated an accuracy of 98.8% for healthy sites, 70.6% for gingivitis sites, and 72.9% for periodontitis sites (Figure 10b).

Results from the 24-month follow-up were integrated into the model to classify site conditions as healthy, gingivitis, or periodontitis based on clinical measurements (GBI and MGI), proinflammatory cytokines, MMPs, and microbial virulence factors at each site. Sites were classified as responders if conditions in periodontitis sites improved and were subsequently reclassified as gingivitis or healthy. Similarly, gingivitis sites that improved were reassigned to healthy status, while healthy sites remained classified as healthy after 24 months. In contrast, sites were designated as non-responders if periodontitis remained unchanged, gingivitis persisted, or deteriorated into periodontitis. Additionally, healthy sites that transitioned to gingivitis or periodontitis were also categorized as non-responders. The test regimen treatment resulted in a greater number of responders (80) compared to the control group (66) and fewer non-responders (34) compared to the control group (75) (Figure 10c). A chi-square test was performed to compare responders and non-responders at 24 months between the test regimen and control treatments, yielding a *p*-value of 0.0002914. This indicates that the test regimen was significantly more effective than the control treatment.

### 2.11. Abundance of Bacteria in Responders and Non-Responders at Baseline and 24-Month Treatment

The gingival sites were categorized into responders and non-responders at the 24-month period based on the model established using baseline results from proinflammatory cytokines, MMPs, virulence factor responses, and the severity of gingivitis and periodontitis. Subsequently, efforts were made to investigate whether bacterial abundance decreased in the responders compared to the non-responders at 24 months.

**Figure 11:**
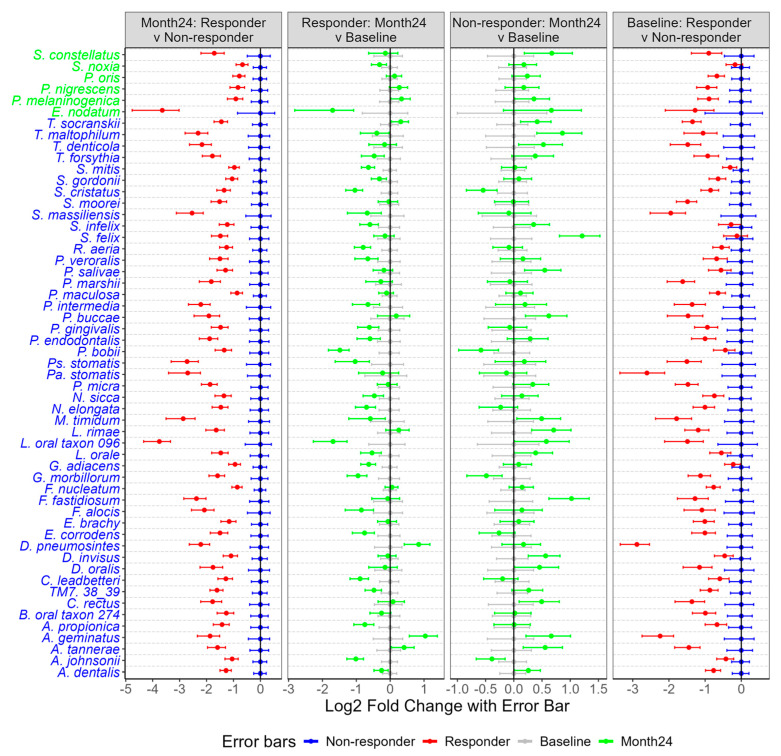
Comparison of bacterial abundance between responders and non-responders at baseline and 24 months in subgingival plaques. Bacteria marked in blue indicate a significant reduction in abundance at 24 months (*p* < 0.01), while bacteria in green show a *p*-value of <0.05 in the comparison of 24 months: responders vs. non-responders. For the remaining three columns (responder: month 24 vs. baseline; non-responder: month 24 vs. baseline; baseline: responder vs. non-responder), only means and standard errors are presented.

Indeed, many bacteria in the responder group exhibited a reduction in abundance at the 24-month follow-up compared to non-responders (Figure 11). Notably, all bacteria in the pink constellation (*F. nucleatum*, *D. invisus*, *T. socranskii*, and TM7_38_39), the red constellation (*T. forsythia*, *T. denticola*, *F. alocis*, and *P. endodontalis*) and *P. gingivalis* showed significant reductions in abundance among responders compared to non-responders (*p* < 0.01). Additionally, bacteria associated with the red constellation (*F. fastidiosum* and *C. rectus*) and the pink constellation (*P. maculosa* and *A. dentalis*) also demonstrated decreased abundance at 24 months in the responder group relative to the non-responder group (*p* < 0.01). At the genus level, *Porphyromonas*, *Tannerella*, *Treponema*, *Filifactor*, *Fusobacterium*, *Dialister*, and *Candidatus Saccharibacteria* (formerly known as *TM7*)—which encompass the core bacterial species in the pink and red constellations—also decreased significantly in the responder group compared to the non-responders at month 24 (*p* < 0.01, Figure S8).

## 3. Discussion

This study provides an in-depth understanding of the subgingival microbiome of healthy, gingivitis and periodontitis sites in 87 subjects with early periodontitis. The baseline analyses show that there is a statistically significant (*p* < 0.05) increase in bacterial DNA, number of bacterial species and Shannon diversity as the periodontal disease condition evolves from health to gingivitis to periodontitis at a site level (Figure 1). This observation is in contrast to a previous report where the amount of bacteria (*p* = 0.165) and Shannon diversity (*p* = 0.156) were not significantly different between healthy, gingivitis, and periodontitis sites [6]. However, that study had a considerably smaller sample size of 21 subjects, which may explain the difference in outcomes. Furthermore, the current study found statistically significantly (*p* < 0.05) increased numbers of subgingival bacterial species implicated in periodontal disease as the disease state progressed from health to gingivitis to periodontitis. These include *F. alocis* (*p* ≤ 0.024), *P. endodontalis* (*p* ≤ 0.017), *T. denticola* (*p* ≤ 0.009), *T. forsythia* (*p* ≤ 0.015), and *P. gingivalis* (*p* ≤ 0.002). *D. invisus* (*p* < 0.001), *F. nucleatum* (*p* < 0.001), *TM7* (*p* ≤ 0.015), and *T. socranskii* (*p* < 0.001) (Figure 2). These results were consistent with Nemoto et al. (2021), where *T. forsythia*, *F. nucleatum*, *T. denticola*, *P. gingivalis*, and *F. alocis* were reported to have increasing abundance in periodontitis sites versus gingivitis sites and gingivitis sites versus healthy sites [6]. With respect to treatment effect in the current study, following 24 months of treatment, the regimen delivered directional reductions in Shannon Diversity (*p* = 0.072) and number of bacterial species (*p* = 0.078) relative to the control treatment. Collectively, these data show that as clinical sites increase in severity from health to gingivitis to periodontitis, a more diverse and complex biofilm is formed, as evidenced by increases in bacterial DNA, number of bacterial species and Shannon diversity. The emergence of bacterial species, including the 8 species highlighted in Figure 2, suggests a likely role in periodontal disease pathogenesis.

This increase in bacterial diversity across healthy, gingivitis and periodontitis sites is paralleled by an increase in bacterial toxins, TLR activation of host cells, and the production of host inflammatory cytokines and enzymes. Specifically, the bacterial toxins gingipain and endotoxin (LPS) are statistically significantly (*p* < 0.001) increased in periodontitis versus gingivitis sites, while PAD is statistically significantly (*p* ≤ 0.001) increased in both periodontitis versus gingivitis sites and gingivitis versus healthy sites (Figure 3). This undoubtedly is related to the emergence of Gram-negative anaerobic bacteria that produce these specific toxins as the disease progresses from health to gingivitis to periodontitis. This increase in bacterial toxins parallels an increase in TLR-ATP and TLR-SEAP activity, with healthy sites having the lowest activity and periodontitis sites having the highest activity (Figure 4). Importantly, 24 months of treatment with the regimen resulted in reductions in TLR-ATP and TLR-SEAP host cell activation values compared to control treatment, with TLR-ATP at month 6 being statistically significant (*p* = 0.027) (Figure 4). TLR receptor activation mediates the initial host inflammatory response by controlling upregulation of IL-1b, which is involved with inducing a broader cytokine cascade [15,16,17]. In the current study, IL-1β was statistically significantly (*p* ≤ 0.001) increased in gingivitis versus healthy sites and periodontitis versus gingivitis sites (Figure 5). Furthermore, other downstream cytokines, including IL-2, IL-8, TNF-α, and IL-13, were also statistically significantly (*p* < 0.05) increased as periodontal disease severity increased, supporting a heightened inflammatory response. Importantly, IL-1β levels were statistically significantly (*p* ≤ 0.001) lower in the regimen group after 24 months of treatment compared to the control group (Figure 5). This observation parallels the clinical gingival bleeding outcomes, where bleeding was statistically significantly (*p* < 0.001) reduced by 39% after 24 months in the regimen versus the control group [15]. Lastly, the host enzymes MMP-1 and MMP-9 were statistically significantly (*p* ≤ 0.003) increased at both periodontitis versus gingivitis sites and gingivitis versus healthy sites (Figure 6). Furthermore, MMP-1 and MMP-9 were statistically significantly decreased at month 24 in the regimen group versus the control group, with MMP-1 (*p* = 0.007) and MMP-9 (*p* = 0.016) (Figure 6). MMP-1 and MMP-9 have both been implicated in mediating periodontal tissue degradation, with MMP-1 demonstrating substrate specificity for Type I, II, III, VII, VIII, X, and XI collagens, gelatin, fibronectin, laminin and tenascin, while MMP-9 demonstrates substrate specificity for Type IV, V, XI, and XIV collagens, gelatin, elastin, laminin and aggrecan [18]. Importantly, elevated levels of IL-1β and MMP-9 are correlated to periodontitis, with higher levels found in periodontitis compared to healthy individuals [19]. Collectively, the data support a reduction in host mediators (e.g., TLR-ATP, IL-1β, MMP-1, and MMP-9) with regimen treatment that are implicated in inflammation and periodontal disease destruction.

Interrogation of the correlations between subgingival bacteria, their toxins, TLR-mediated virulence response, host cytokines and enzymes, and clinical endpoints established key relationships that exist between these entities. Endotoxin, PAD, TLR_ATP, and TLR-SEAP demonstrated the strongest associations to specific groups of bacteria, while IL1-β, MMP-1, MMP-9, MGI, and GBI also demonstrated a strong association to those same bacteria (Figure 7). Bacterial toxins were found to have a statistically significant (*p* < 0.05) association with both TLR-ATP and TLR-SEAP activation, with endotoxin having correlations of 0.65 and 0.78, respectively, and PAD having correlations of 0.59 and 0.76, respectively (Figure 8). In addition, IL-1β, the keystone cytokine mediator of inflammation, was statistically significantly (*p* < 0.05) associated with endotoxin (r = 0.45), PAD (r = 0.62), MMP-1 (r = 0.51), and MMP-9 (r = 0.77). Furthermore, apparent networks of bacteria that were moderately correlated to the host biomarker amount were identified. *F. alocis* and *P. endodontalis* were consistently associated with the red complex bacteria *T. denticola* and *T. forsythia*, suggesting that they are potentially important pathogens in periodontal disease (Figure 8). In addition, *T. socranskii*, *TM7*, and *D. invisus* were associated with the orange complex bacteria *F. nucleatum*. Lastly, there were a number of bacteria identified that were not correlated to a host biomarker response, suggesting a role in the maintenance of periodontal health. These include bacteria in the genera *Rothia* (*R. mucilaginosa)*, *Haemophilus* (*H. haemolyticus* and *H. paraphrohaemolyticus*), *Streptococcus* (*S. mutans* and *S. sobrinus*), *Enterobacter* (*E. hormaechei*) and *Neisseria* (*N. cinerea)* (Figure 9).

To further explore the predictive nature of biomarkers as a measurement of treatment effectiveness in differentiating regimen from control treatment, a random forest model was created using the baseline levels of biomarkers and clinical presentation observed in healthy, gingivitis, and periodontitis sites. The accuracy of this model was predominantly driven by gingival bleeding, followed by TLR activation (e.g., TLR-ATP, TLR-SEAP) and toxin level (e.g., PAD, endotoxin). The model accurately predicted baseline disease state at a site level with a 98.8% accuracy for health, a 70.6% accuracy for gingivitis and a 72.9% accuracy for periodontitis (Figure 10). The absence of bleeding likely drives the extraordinarily high accuracy in the healthy group. Given that both gingivitis and periodontitis sites required the presence of bleeding to be classified, bleeding is not responsible for driving the accuracy of these classifications. This suggests that the observed >70% accuracy is based on a differentiated integrated biomarker response. Furthermore, the integrated random forest model was able to differentiate the regimen from the control at 24 months, with the regimen having statistically significantly (*p* < 0.001) less disease than the control group. These results are consistent with the clinical outcomes where whole-mouth gingival bleeding and pocket depth were measured. Importantly, the clinical outcomes are based on measuring 6 sites per tooth and up to 168 sites per mouth, while the random forest model is based on measuring only 9 sites per mouth in a more detailed fashion that involves both clinical measures and bacterial and host biomarkers. It is remarkable that 9 sites interrogated in a more comprehensive manner can provide a similar differentiation in clinical outcomes as compared to measuring all sites in the mouth (up to 168) via clinical examination. This recognition that evaluating a limited subset of sites in a more comprehensive manner (e.g., clinical bleeding, bacterial toxins, host TLR activation, host cytokines and enzymes) can differentiate treatment outcomes with similar fidelity to measuring whole-mouth clinical outcomes is potentially quite powerful. It has implications for clinical study design and monitoring of patient outcomes in dental practice.

**Figure 12:**
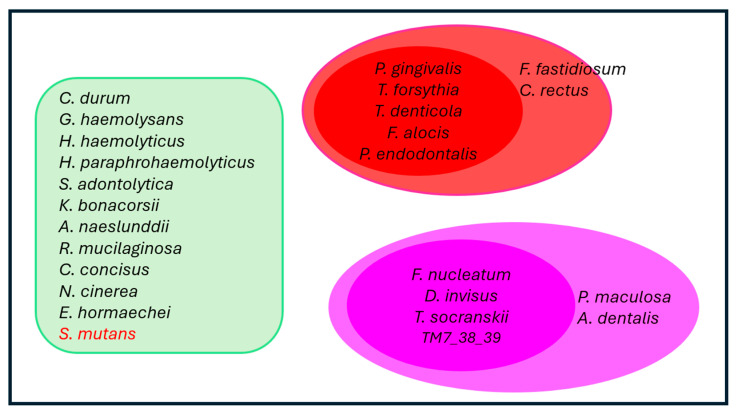
Bacterial Networks in Subgingival Plaques. This figure depicts the clustering of bacteria based on their correlations with nine statistically significant factors that distinguish healthy gingival conditions from gingivitis and periodontitis. The identified factors include proinflammatory cytokines (IL-1β), matrix metalloproteinases (MMP1 and MMP9), bacterial virulence factors (such as endotoxins, gingipains, and PAD), TLR responses (TLR-SEAP and TLR-ATP), as well as clinical symptoms associated with gingivitis and periodontitis (GBI and MGI). The most strongly correlated bacteria (*p* < 0.001) are grouped into two distinct clusters, represented by ovoid shapes in the red and orange areas. Within these clusters, all bacteria exhibit a correlation coefficient of 0.6 or higher. Notably, *P. gingivalis* is an exception to this trend (see Figure 8 for additional details). Bacteria located near the red or orange ovoids also demonstrate correlation coefficients of 0.6 or greater with 3 or all members of their respective clusters (Figure S4 for more details). In contrast, bacteria in the light green area do not show significant correlations with four or more of the nine listed factors (*p* > 0.05). Importantly, all bacteria within the green box, except for *S. mutans*, exhibit weak correlations with one another. *S. mutans*, in particular, shows a negative correlation with other bacteria in subgingival plaques (Figure 9 for more details).

Further, the random forest model was used to distinguish bacterial species overabundance in non-responder versus responder sites at month 24. A number of species were found to be overabundant in non-responders versus responders, including *T. forsythia*, *T. denticola*, *F. alocis*, *P. endodontalis*, *F. fastidiosum*, *C. rectus*, *F. nucleatum*, *D. invisus*, *T. socranskii*, *TM7*, *P. maculosa*, *P. oris*, *P. nigrescens*, and *L. rimae*. Taken collectively, these data were used to revisit Socransky’s red and orange complex construct, leading to an update of these bacterial networks based specifically on learnings from the patient population in this study (Figure 12). Socransky, in his seminal publication, acknowledged the limitations of his study, characterizing it as “an initial attempt at evaluating inter-relationships among subgingival species” [4,20]. Recently, research scientists have updated the original Socransky bacterial complexes, creating emerging evidence to support the identification of new bacterial clusters [20,21]. The data presented in this research justifies the inclusion of *F. alocis* and *P. endodontalis* in the red complex with *T. denticola* and *T. forsythia*, as well as *T. socranskii*, *D. invisus* and *TM7* in the orange complex with *F. nucleatum*. Supporting this belief, other researchers have similarly identified *F. alocis* as an important periodontal pathogen [22,23,24]. *F. alocis* demonstrates a synergistic partnership in terms of growth with *F. nucleatum* and forms heterotypic complexes with *P. gingivalis* [22]. Importantly, *F. alocis* has been shown to promote alveolar bone loss, as well as overexpression of proinflammatory cytokines in a mouse periodontal infection model [23]. These effects were abolished in TLR-2-deficient mice, implying *F. alocis’*s effects on inflammation and alveolar bone loss were mediated through TLR activation. Furthermore, a recent meta-taxonomic study based on V3–V4 16S RNA sequencing used bioinformatics to group unambiguous taxon groups and reported that *P. gingivalis*, *P. endodontalis*, *F. alocis*, *T. denticola*, *T. forsythia*, *F. fastidiosum*, and *C. rectus* were clustered into a network that was associated with periodontal disease [20].

Strengths of this experiment include its design, which incorporated subgingival plaques from healthy, gingivitis, and periodontitis sites across all participants, thereby minimizing variation among individuals. Another strength was the large sample size, with a total of 498 samples sequenced using shotgun procedures. Additionally, the study effectively measured the gingival inflammatory environment by assessing proinflammatory cytokines and MMPs. Finally, it evaluated virulence factors using both enzymatic and cell-based assays. These comprehensive datasets enabled the construction of networks linking bacteria, clinical measurements, proinflammatory cytokines, TLR responses, and MMPs.

There are a few potential limitations to this research. The availability of complete genome sequences for oral bacteria is currently limited, which affects the accuracy of taxonomic classification. Additionally, this study did not incorporate proteomics or metabolomics, both of which could provide valuable insights for differentiating between healthy, gingivitis, and periodontitis sites within participants. Furthermore, some sequences may misclassify certain bacteria. It is important to note that the red and pink constellations were constructed based on correlation rather than direct visualization. Experimental verification of the spatial arrangement of bacteria within these constellations using techniques such as fluorescence in situ hybridization is still recommended.

## 4. Materials and Methods

The study compared an oral health regimen (Oral-B oscillating–rotating power toothbrush, Oral-B 0.454% SnF_2_ toothpaste, Crest 0.07% CPC mouthrinse, Oral-B dental floss (all Procter & Gamble, Cincinnati, OH, USA) and dental prophylaxis every 6 months) to usual care/control (manual toothbrush, 0.245% NaF toothpaste and dental prophylaxis every 6 months) on the subgingival microbiome over 24 months. The clinical study protocol was approved by an Institutional Review Board (Ref# 0482-13-HMO, Hadassah Medical Organization Helsinki Committee, Jerusalem, Israel) on 24 December 2013, and the study was registered in the ISRCTN database (ISRCTN66780304). Written informed consent was obtained for each participant prior to participation in the study.

### 4.1. Subgingival Sample and GCF Collection

GCF and subgingival plaque samples were collected from participants at baseline and at 6, 12, 18, and 24 months, targeting three sites each from healthy, gingivitis, and periodontitis categories. Prior to collection, the target site was isolated with cotton rolls and dried with a gentle stream of air to eliminate saliva contamination. Supragingival plaque was carefully removed to ensure accurate sampling. GCF was obtained by inserting a periostrip (Oraflow Inc., Lynbrook, NY, USA) 1–2 mm into the pocket for 30 s. The periostrip was then cut to approximately 8 mm in length and placed into a 1.5 mL sterile tube containing 200 μL of phosphate-buffered saline (PBS). For subgingival plaque sampling, a Universal Barnhart 5/6 curette was gently inserted into the pocket without applying pressure to minimize dislocation of the plaque. A single vertical stroke was performed upon encountering tissue resistance at the apical part of the pocket. All samples were vortexed, placed on dry ice, and stored at −80 °C until shipping on dry ice. During storage, samples were maintained in an ultracold freezer at −70 °C at the Mason Business Center until analysis.

### 4.2. Subgingival Sample and GCF Processing

Upon analysis, samples were thawed on ice and vortexed for 5 s to achieve homogeneity. The periostrip was carefully removed using sterile forceps. The sample tubes were then centrifuged in a refrigerated microcentrifuge at 14,000 rpm for 10 min at 4 °C, and the supernatant (GCF) was collected for further analysis. To the pellet in each sample tube, 180 μL of Dulbecco’s Phosphate-Buffered Saline (DPBS) without calcium and magnesium (Thermo Fisher Scientific, Waltham, MA, USA) was added and vortexed to ensure thorough dispersion. For DNA sequencing, half of this solution (90 μL) was transferred to a separate microtube. Samples from each subject representing 3 healthy, 3 gingivitis, and 3 periodontitis conditions at each time point were pooled to create composite samples for healthy, gingivitis, and periodontitis conditions, ensuring sufficient DNA for sequencing at Global Bioscience Genomics Lab in Mason. Each subject provided six distinct samples, with three samples representing healthy sites, gingivitis, and periodontitis at baseline, and the other three collected at month 24. A total of 83 subjects—38 treated with the regimen and 45 in the control group—had all six samples available, resulting in a total of 498 samples for sequencing. For gingipain, endotoxin, PAD, TLR-ATP, TLR-SEAP, cytokines, and MMPs, each of the nine sites per subject was analyzed individually, and means and medians were calculated for healthy, gingivitis, and periodontitis conditions.

### 4.3. Sonication of Subgingival Plaques

The remaining half of each sample (90 μL) was processed individually for virulence analysis. To each sample, an additional 210 μL of DPBS was added, resulting in a final volume of 300 μL. Samples were sonicated using an eight-element ultrasonic probe (Vibracell 600 probe sonicator, Sonics & Materials, Inc., Newtown, CT, USA) housed within a sound-deadening enclosure in a chemical fume hood. The sonicator was calibrated according to the manufacturer’s specifications and set to 30% amplitude with a pulse sequence of 1 s on and 2 s off for a total duration of 3 min. Following sonication, samples were centrifuged at 12,000 rpm in a microcentrifuge at 4 °C for 10 min, and the supernatants of the subgingival plaque samples were collected for subsequent virulence analysis.

### 4.4. PAD Detection

PADs are a group of enzymes that catalyze the conversion of protein-bound arginine residues to citrulline, a process known as citrullination. In humans, there are five distinct isoforms of PADs (PAD1, 2, 3, 4, and 6). In the context of GCF, PADs are sourced from both gingival tissue and subgingival biofilm. Notably, the PADs produced by the bacterium Porphyromonas gingivalis are referred to as Porphyromonas PADs (PPADs). To assess the activity of peptidylarginine deiminases from *P. gingivalis* (PPAD) and human gingival tissue (PAD), the PAD4 Inhibitor Screening Assay Kit (Cayman, Ann Arbor, MI, USA) was utilized. The assay employed N-α-benzoyl-L-arginine ethyl ester (BAEE) as a substrate, which is catalyzed by both human PADs and bacterial PPADs, resulting in the release of ammonia. This ammonia subsequently reacted with a detection substrate to produce a fluorescent compound. Ammonium chloride (NH_4_Cl) was used to create a standard curve for quantification. The assay was conducted according to the manufacturer’s instructions. In brief, a sample of the standard curve and tested samples was added to each well of a 96-well plate, followed by the addition of the reaction mix. The plates were incubated at 37 °C for 30 min. The PAD reaction was then halted using a stop solution provided in the assay kit, after which an ammonia detection mixture was added, and the plates were incubated for an additional 15 min. Fluorescence was measured using a multi-modal microplate reader (SpectraMax iD3, Molecular Devices Corporation, San Jose, CA, USA) at an excitation wavelength of 410 nm and an emission wavelength of 480 nm. The fluorescence intensity data were converted into RFUs. One unit of activity was defined as the fluorescence produced by 1 µM NH_4_Cl when reacted with the detection solution.

### 4.5. Gingipain Enzymatic Assay

Gingipains are trypsin-like proteinases produced by *P. gingivalis* and are classified as cysteine proteinases. This group includes three primary types: lysine-gingipain (Kgp), arginine-gingipain A (RgpA), and arginine-gingipain B (RgpB). A specific substrate for these enzymes, FITC-Arg-D-Lys-Lys-Dabcyl, was identified by Kaman et al. in their research on detecting *P. gingivalis* in the diagnosis of periodontitis [25]. In this substrate, the fluorescence of FITC is quenched by Dabcyl due to their proximity. Upon cleavage of the bond at Arg-D-Lys, FITC and Dabcyl are separated, allowing FITC to emit fluorescence upon excitation. This unique fluorescent property enables the quantification of gingipain activity in gingival crevicular fluid (GCF). The assay was conducted following the methodology established by Kaman et al. [25], with modifications. The reaction mixture consists of Tris-HCl (pH 7.5), L-cysteine, and FITC-Arg-D-Lys-Lys-Dabcyl. Initially, samples are aliquoted into a 96-well plate, followed by the addition of the reaction mixture. The plates are then covered with a transparent plastic sealing cover and centrifuged quickly at 1200 rpm for 30 s using a swing adaptor, ensuring that the reaction volume settles at the bottom. After centrifugation, the plates are vortexed at high speed (setting 10) for 30 s to thoroughly mix the reagents, then incubated at 37 °C. Fluorescence readings are taken after 24 h of incubation using a multi-modal microplate reader (SpectraMax iD3, Molecular Devices Corporation, San Jose, CA, USA) with an excitation wavelength of 485 nm and an emission wavelength of 530 nm.

### 4.6. Endotoxin Measurement in Subgingival Plaques

Endotoxins in subgingival plaques were quantified using the Pierce Limulus Amebocyte Lysate (LAL) chromogenic endotoxin quantitation kit (Thermo Fisher Scientific, Waltham, MA, USA). This assay specifically detects LPS, which catalyzes the activation of a proenzyme in the modified LAL system. Upon activation, the proenzyme cleaves p-Nitroaniline (pNA) from the colorless substrate Ac-Ile-Glu-Ala-Arg-pNA, resulting in a measurable product that is photometrically assessed at 405 nm. To evaluate the activities of bacterial components that activate the proenzyme, ninety-six-well microplates were equilibrated in a heating block at 37 °C for 10 min. Given the high concentration of endotoxins present in subgingival plaque, pooled samples were serially diluted to determine the optimal dilution for LAL activity, which was identified as a 1:200 dilution (1 μL of subgingival plaque supernatant mixed with 199 μL of endotoxin-free water). Subsequently, 50 μL of each standard and diluted plaque sample were added to the microplate wells. LAL reagent, chromogenic substrate, and stop solution were then sequentially added according to the manufacturer’s instructions. The plates were gently shaken, and absorbance was measured at 405 nm using a Spectramax M3 plate reader (Molecular Devices, San Jose, CA, USA). A standard curve was constructed using the *E. coli* endotoxin standard provided in the kit, allowing for the calculation of endotoxin concentrations expressed in endotoxin units per milliliter (EU/mL), where one endotoxin unit/mL corresponds to approximately 0.1 ng of endotoxin in 1 mL of solution. Results were recorded as relative EU.

### 4.7. TLR2-SEAP Assay

The HEK-Blue™-hTLR2 cell line, sourced from InvivoGen (San Diego, CA, USA), is designed to express human TLR2 and SEAP transgenes. The SEAP reporter gene is under the control of the IFN-β minimal promoter, which incorporates five binding sites for NF-κB and AP-1. Ligand binding to the TLR2 receptor activates these transcription factors, resulting in SEAP production. To further enhance the TLR2 response, the CD14 co-receptor gene was also transfected into the cells. CD14 binds to LPS but lacks an intracellular component and is, thus, incapable of signaling. HEK-Blue™-hTLR2 cells were cultured in T75 flasks with 15 mL of growth medium composed of DMEM supplemented with 10% fetal calf serum and maintained at 37 °C with 5% CO_2_ and 95% humidity for three days. For the assay, 10,000 cells were seeded into each well of a 96-well plate with 100 µL of growth medium and incubated for 72 h under the same conditions until day 4 [26]. On day 4, 90 µL of the medium was replaced with HEK-Blue™ Detection medium (InvivoGen, San Diego, CA, USA), which develops a purple/blue color in the presence of alkaline phosphatase, the product of the reporter gene. Following this, 10 µL of subgingival plaque samples were added to the wells, and the cells were incubated for an additional 19 h. The expression of the SEAP reporter gene was quantified by measuring absorbance at 625 nm using a Spectramax M3 plate reader (Molecular Devices, Sunnyvale, CA, USA).

### 4.8. TLR-ATP Biosensor Assay

The TLR-ATP biosensor cell line was developed in collaboration with TempoBiosciences (San Francisco, CA, USA) and incorporates two key components: the TLR2 receptor and an ATP biosensor. HEK-Blue™-hTLR2 cells, sourced from Invivogen (San Diego, CA, USA), were created by co-transfecting HEK293 cells with the human TLR2 receptor and additional genes. The fluorescent ATP biosensor, TempoATP™ [27], was integrated into the HEK293 TLR2 receptor cell line. This biosensor assay provides rapid kinetics, enabling the measurement of real-time cellular ATP metabolism and oxidative phosphorylation—both directly linked to mitochondrial health and cellular bioenergetics. The TempoATP™ biosensor is a fusion protein composed of an ATP-binding peptide sequence and a green fluorescent protein (GFP) sequence. This fusion protein is constitutively expressed in the cells. As intracellular ATP levels rise, ATP binds to the biosensor, inducing a conformational change that alters the structure of the GFP domain. This structural change enhances the fluorescence of the GFP, with fluorescence intensity reflecting ATP presence—becoming brighter or dimmer depending on the binding event of the fusion protein to ATP. Fluorescence changes were measured in real time using the IncuCyte^®^ Live-Cell Analysis System (IncuCyte Zoom, Sartorius AG, Ann Arbor, MI, USA), with an excitation wavelength of 519 nm and an emission wavelength of 535 nm (green fluorescence). This setup enabled continuous monitoring of fluorescence changes as they occurred.

For cell culture, 500,000 TLR-ATP biosensor cells were seeded in 15 mL of growth medium, which consisted of DMEM supplemented with 10% fetal calf serum, 1× HEK-Blue™ Selection (a combination of selective antibiotics), 50 μg/mL Normocin, and 1 μg/mL Puromycin (Thermo Fisher Scientific, Waltham, MA, USA) in T75 flasks. The cells were incubated for three days at 37 °C with 5% CO_2_ and 95% humidity. For treatment, 7500 cells were plated per well in a 96-well plate with 100 µL of growth medium and incubated for an additional 72 h under the same conditions. On day 4, the growth medium was replaced with HEK-Blue™ Detection medium (Invitrogen, San Diego, CA, USA), and subgingival plaque samples were added to the wells. Results were processed and analyzed using the IncuCyte^®^ S3 Software provided with the instrument. Data processing and analysis were conducted using the IncuCyte^®^ S3 Software accompanying the instrument. Lipopolysaccharide from *P. gingivalis* (LPS-PG), sourced from InvivoGen (San Diego, CA, USA), served as the assay standard, with concentrations ranging from 0.01 to 3 μg/mL. The virulence factors present in the subgingival plaques were quantified as LPS-PG equivalents in μg/mL.

To estimate the LPS equivalent based on the intensity of the IncuCyte measurements, a regression model was developed to relate the onset time to LPS concentrations in the assay. Onset time is defined as the moment when the Area Under the Curve (AUC) of the IncuCyte measurements reaches 30% of its saturation value. The AUC was calculated using the ‘auc’ function from the MESS library in R (version 4.0.1), while onset time was estimated using the ‘approx’ function in R based on the AUC plot. The logarithm of LPS concentration was fitted to the inverse of onset time using the equation:

log(LPS conc) = a × (1/Onset time) + b

This model yielded an (R^2^) value of 0.995 when applied to standard test samples with known LPS concentrations (33 plates with 96 wells each). The regression model was subsequently used to estimate the LPS concentrations of the investigated samples based on their onset times.

### 4.9. Cytokine and MMP Measurement

Cytokine and MMP levels were assessed using the V-PLEX Proinflammatory Panel 1 Human Kit, which includes the measurement of IFN-γ, IL-1β, IL-2, IL-4, IL-6, IL-8, IL-10, IL-12p70, IL-13, and TNF-α. Additionally, the Human MMP 3-Plex Ultra-Sensitive Kit was employed to measure MMP-1, MMP-3, and MMP-9. Both kits were obtained from Meso Scale Diagnostics (Rockville, MD, USA). GCF samples were diluted at a ratio of 1:200 in phosphate-buffered saline (PBS), as described in Section 4.2. An equal volume of 10 µL of the diluted GCF samples was then added to each well of the assay plate. All subsequent assay procedures were performed in accordance with the manufacturer’s instructions. Concentrations of the measured cytokines and MMPs were reported in picograms per milliliter (pg/mL).

### 4.10. DNA Extraction

DNA extraction was performed using the DNAdvance Kit (A48705, Beckman Coulter Life Sciences, Indianapolis, IN, USA), with modifications to the manufacturer’s protocol. A detailed description of the DNA extraction method will be available upon request. The extractions were carried out in a 96-well plate format, with sample locations randomized to minimize potential biases. The extraction procedure included several key steps: bead beating, protein digestion, and DNA purification using magnetic beads. The addition of magnetic beads, along with subsequent ethanol washes and elution steps, was automated using the Beckman FX robot (Beckman Coulter Life Sciences, Indianapolis, IN, USA). Following extraction, the quantity of DNA was measured using the Thermo Scientific Varioskan LUX Multi-mode Microplate Reader in conjunction with the Qubit 1X dsDNA HS Assay Kit (Q3323, Thermo Fisher, Hanover Park, IL, USA).

### 4.11. Metagenomic Sequencing

Extracted DNA samples were normalized to a concentration of 0.2 ng/μL and randomized across sequencing plates. The DNA libraries were prepared using Illumina’s Nextera XT DNA Library Preparation Kit (FC-131-1096, Illumina, San Diego, CA, USA) according to the manufacturer’s instructions. Library purification and size selection were performed using 0.6× AMPure XP Beads (A63881, Beckman Coulter Life Sciences, Indianapolis, IN, USA). The resulting DNA libraries were quantified with the Qubit dsDNA HS Assay Kit (Q3323, Thermo Fisher, Hanover Park, IL, USA) and assessed for size distribution on the Agilent Bioanalyzer with the High Sensitivity DNA Kit (5067-4626, Agilent Technologies, Santa Clara, CA, USA), confirming fragment sizes ranged from 250 to 1000 bp. The libraries were then normalized to 2 nM, and equal volumes were pooled. To serve as an internal sequencing control, PhiX DNA (FC-110-3002, Illumina, San Diego, CA, USA) was added at a concentration of 1%. Finally, the pooled libraries were sequenced on the NextSeq 500 instrument using the NextSeq 500/550 High Output Kit v2.5 (300 Cycles), with a paired-end read configuration of 2 × 150 bp (Illumina, San Diego, CA, USA).

### 4.12. Bioinformatics Analysis

Sequence bases with quality scores below 30 were discarded from both the 3′ and 5′ ends using cutadapt version 4.0. The remaining reads were further filtered by samtools version 1.21, bowtie2 version 2.5.1, and SNAP version 2.0.3 to remove reads mapped to the human genome (GRCh38) and Phix genome. Read pairs with a read mean quality score below 30 or shorter than 75% of the read length (i.e., 105 bp) were also discarded. FastQC v0.12.1 was performed on the remaining reads (Pass Filter Reads). Pair-end FASTQ files were separately merged in case multiple read files were used for the same sample. Samples with number of sequence reads less than the negative controls were discarded. Analysis of the taxonomic distribution was performed by MetaPhlAn4.06, a profiling algorithm using clade-specific marker genes, and Kraken2 version 2.1.3, a K-mer-based algorithm with an expanded database including ~3000 fungal genomes from NCBI. Gene and pathway analysis were performed on HUMAnN 3.8. To further check the taxonomy classification and gene classification for oral bacteria, cleaned reads were aligned to a database made from the 83 oral bacterial transcriptomes by Salmon v1.10.0. The counts of sequence reads were normalized based on the sample’s total detected read counts as relative abundance for statistical analysis. The relative abundance of microbial species was utilized for further analysis. To determine microbial biomass from the plaques, absolute abundance was calculated by multiplying relative abundance by the total microbial DNA extracted from the plaque samples. Given that the purified DNA from the plaques contains both human and microbial DNA, microbial DNA was estimated by multiplying the total DNA amount from each sample by the ratio of microbial sequences to total sequences obtained.

### 4.13. Statistical Analysis

All statistical analyses were done using R Statistical Software (v4.4.3). Shannon diversity was assessed using the alpha function from the R package microbiome (v1.26.0). Paired *t*-tests were employed for comparisons involving dependent samples, such as those comparing different healthy status groups at baseline and month 24. Independent *t*-tests were conducted to compare the treatment regimen to the control group at each time point. Those *t*-tests were used on microbial DNA, Shannon diversity, and the number of species. For correlation analysis involving repeated measurements, the rmcorr function from the corresponding R package (v0.7.0) was utilized, which is specifically designed for analyzing repeated measures like multiple samples from the same subject. A root transformation was applied (taking the 10th root) to mitigate skewness prior to analysis. Correlation plots were generated using the corrplot function from the R package (v0.95) of the same name, while network visualizations were created using functions from the igraph package (v2.1.4). A Random Forest model was constructed using the randomForest function from the respective R package (v4.7.1.2), with a seed set to 12,345 and the number of trees parameter configured to 400. Variables with a feature importance score below 2 were excluded from the model. To model the microbiome data, a zero-inflated Gamma distribution with subject as a random effect was applied using the glmmTMB function from the corresponding package (v1.1.11), and the effects of Time and Responder categories were evaluated with the emmeans package (v1.11.2.8). Pearson’s chi-squared test was performed to assess the relationship between responder categories and treatment groups using the chisq.test function in R. Biomarker endpoints, including endotoxin, gingipain, PAD, TLR2-SEAP, TLR-ATP, cytokines, and MMP measurements, were not normally distributed, so the non-parametric method was applied to all the biomarker endpoints. The Wilcoxon Signed Rank test was used to compare dependent samples, including various healthy status groups (healthy sites, gingivitis sites, and periodontitis sites) at baseline using wilcox.test from rstatix package (v0.7.3) in R. Additionally, Kruskal–Wallis test followed by Dunn’s test was utilized to compare treatment regimen to the control group at each time point, using kruskal.test and dunn_test functions from rstatix package in R. Summary statistics (mean and standard errors) were reported for endotoxin, gingipain, PAD, cytokines, and MMP measurements in Figure 3, Figure 5 and Figure 6 with the exception that the median was reported for TLR2-SEAP and TLR-ATP in Figure 4. All *p*-values are reported without adjustments for multiple comparisons, with *p*-values less than 0.001 denoted as <0.001. Only significant *p*-values less than or equal to 0.05 are presented, with a few exceptions made for *p*-values between 0.05 and 0.1, which are also reported.

### 4.14. Construction of Bacterial Network

For pathogenic bacterial networks, bacterial species were selected using two filtering criteria on the correlation coefficients and *p*-values between the nine factors and bacterial species. The *p*-value threshold was set at 0.001, ensuring a high level of statistical significance. For the correlation coefficients, various thresholds were used to select approximately 20 bacterial species that satisfied all filtering conditions. The resulting bacterial species, along with the nine factors, were shown in the pathogenic bacterial network. Connections within the network were established only if the correlation coefficient exceeded 0.6, indicating a strong positive correlation.

For non-pathogenic, or commensal, bacterial networks, different selection criteria were employed. Bacterial species were included if at least six of their correlations with the nine factors were negative. Additionally, any bacteria named by numerical identifiers were excluded from the final list. Only significant correlations were displayed in the correlation heatmap and network plot, emphasizing the most relevant relationships within the commensal bacterial networks.

## 5. Conclusions

The data from this study establishes that healthy, gingivitis and periodontitis sites are differentiated by the amount and diversity of bacteria in subgingival biofilm, amount of bacterial toxin, amount of TLR activation, and amount of host-derived cytokine and enzymes. Following the 24 months of treatment with the regimen, the regimen was found to directionally reduce bacterial amount and diversity while also statistically significantly reducing critical biomarkers (IL-1β, MMP-1, MMP-9) associated with inflammation and periodontitis. Furthermore, the random forest model based on a combination of clinical endpoints, bacterial toxins, host TLR activation and host cytokines and enzymes also found the regimen treatment to be more effective in reducing disease than the control treatment in this early periodontitis population. This supports the potential importance of an oscillating–rotating power toothbrush, SnF_2_ dentifrice and CPC mouthrinse used in combination as an effective adjunctive therapy in the maintenance of periodontal disease.

## Figures and Tables

**Table 1:** Baseline demographic and clinical characteristics of the clinical population (adapted with permission from the *Canadian Journal of Dental Hygiene* [15]).

Demographic or Clinical Characteristic	Control (*n* = 53)	Regimen (*n* = 54)	Overall (*N* = 107)	*p*-Value
Age (years)				
Mean (SD)	47.6 (10.29)	45.9 (10.79)	46.7 (10.53)	0.4075 ^a^
Min–max	28–64	29–63	28–64	
Sex				
Female ^b^, *n* (%)	26 (49)	27 (50)	53 (50)	1.0000 ^c^
Male ^b^, *n* (%)	27 (51)	27 (50)	54 (50)	
Tobacco				
No ^b^, *n* (%)	46 (87)	46 (85)	92 (86)	1.0000 ^c^
Yes ^b^, *n* (%)	7 (13)	8 (15)	15 (14)	
Mean Number of Gingival Bleeding Sites (SD)	56.11 (14.921)	55.36 (13.723)	55.73 (14.266)	0.784
Mean Modified Gingival Index score (SD)	1.69 (0.154)	1.68 (0.129)	1.68 (0.141)	0.723
Mean Probing Pocket Depth, mm (SD)	2.25 (0.186)	2.25 (0.206)	2.25 (0.195)	0.980
Mean Clinical Attachment Level, mm (SD)	2.62 (0.228)	2.60 (0.241)	2.61 (0.234)	0.783
Mean Gingival Recession, mm (SD)	0.36 (0.174)	0.35 (0.139)	0.36 (0.157)	0.741

^a^ Two-sided ANOVA *p*-value for the treatment comparison. ^b^ The number (percent) of participants in each category. ^c^ Two-sided Fisher’s exact test *p*-value for the treatment comparison.

## Data Availability

Metagenomics Data is deposited in the NCBI SRA Database (BioProject ID: PRJNA1346422, Submission ID: SUB15710265).

## Supplementary Materials

The following supporting information can be downloaded at https://www.mdpi.com/article/10.3390/ijms262210823/s1.
