# Supplementary material for: Identification of Bacterial Networks and Relationship to Host Responses in Early Periodontitis Population over 24 Months
Source: Int J Mol Sci. 2025 Nov 7;26(22):10823. doi: 10.3390/ijms262210823 (PMC12652036; doi:10.3390/ijms262210823)

Supplemental Materials

Identification of Bacterial Networks and Relationship to Host Responses in Early Periodontitis Population over 24 Months

Aaron R. Biesbrock [1,*], Sancai Xie [2], Ping Hu [2], Cheryl S. Tansky [3], Xingtao Wei [2], Hao Ye [1], Benjamin Circello [2], Avi Zini [4], Guy Tobias [4], Makio Tamura [2] and Mirjana Parlov [2]

Figure S1. Venn diagrams of identified bacterial species in healthy, gingivitis and periodontitis sites at baseline and month 24.

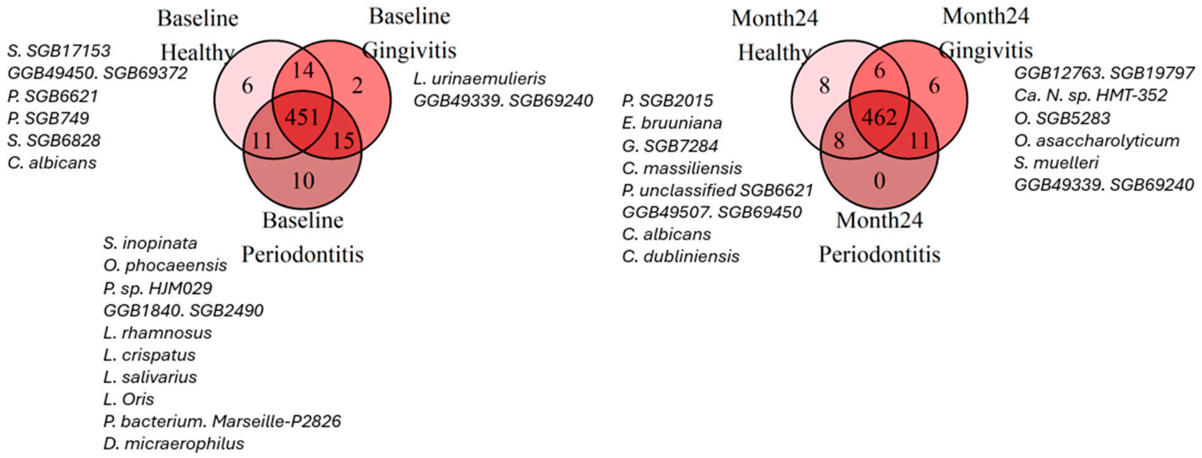

Bacteria were plotted separately at baseline and month 24. Bacteria unique to healthy, gingivitis, and periodontitis sites were listed alongside their respective classifications.

Figure S2. ***P. gingivalis*** **in healthy, gingivitis and periodontitis sites in subgingival plaques at baseline.**

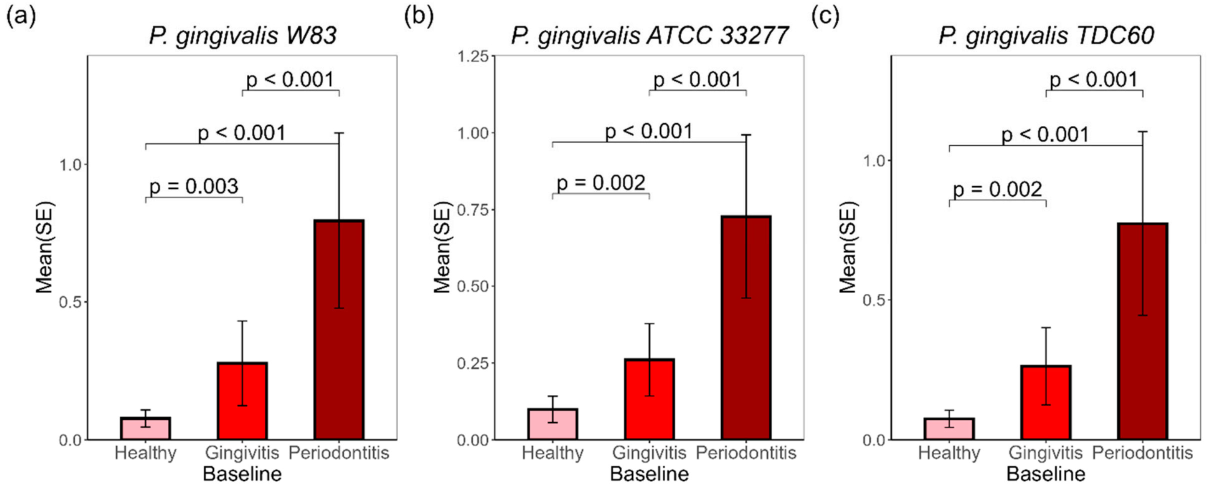

A customized oral genome library with genes from three *P. gingivalis* strains—*W83, ATCC33277, and TDC60*—were mapped the genomic sequences of this study to calculate relative percentages and absolute amounts (Salmon method). Bar plots were constructed to represent the means and SE.

Figure S3. Correlation between the core bacteria in the red and pink constellations and other bacteria in subgingival plaques at baseline with a correlation coefficient equal to or greater than 0.5.

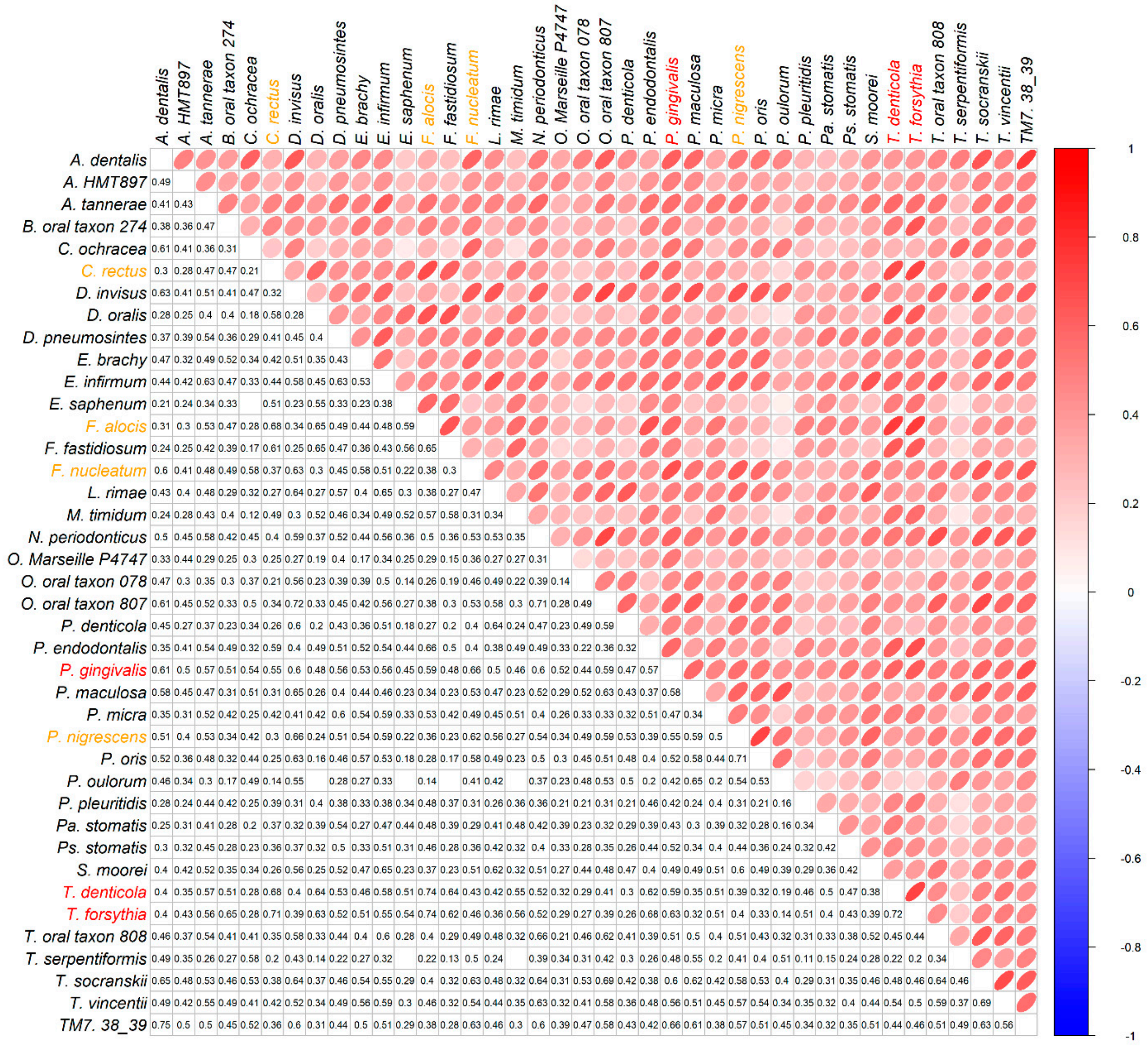

Figure S4. Networks between the core bacteria in the red and pink constellations and other bacteria in subgingival plaques at baseline with a correlation coefficient equal to or greater than 0.5.

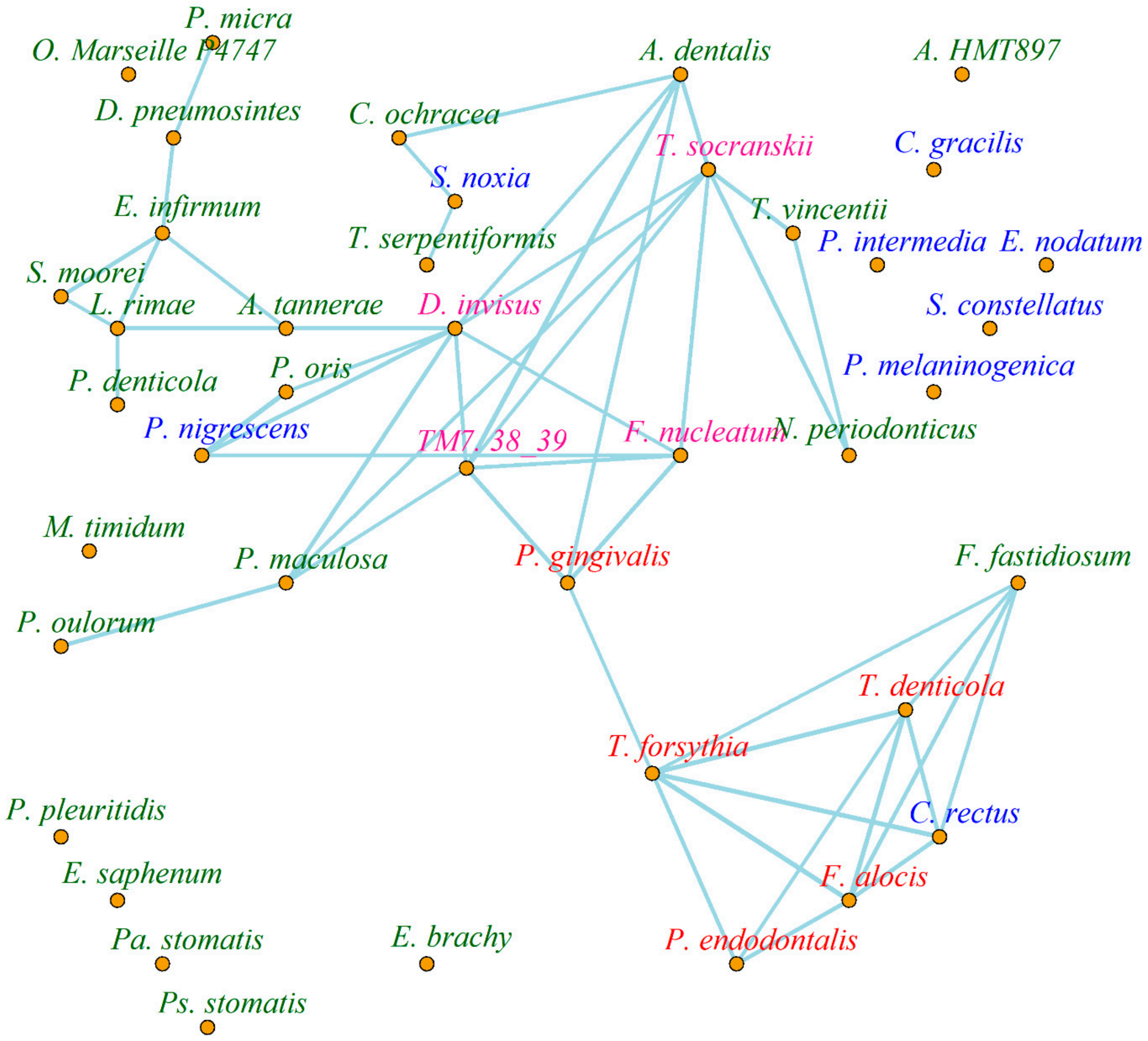

Figure S5. Bar plots of bacteria that were not correlated with gingivitis and periodontitis measurements in healthy, gingivitis and periodontitis sites in subgingival plaques at baseline.

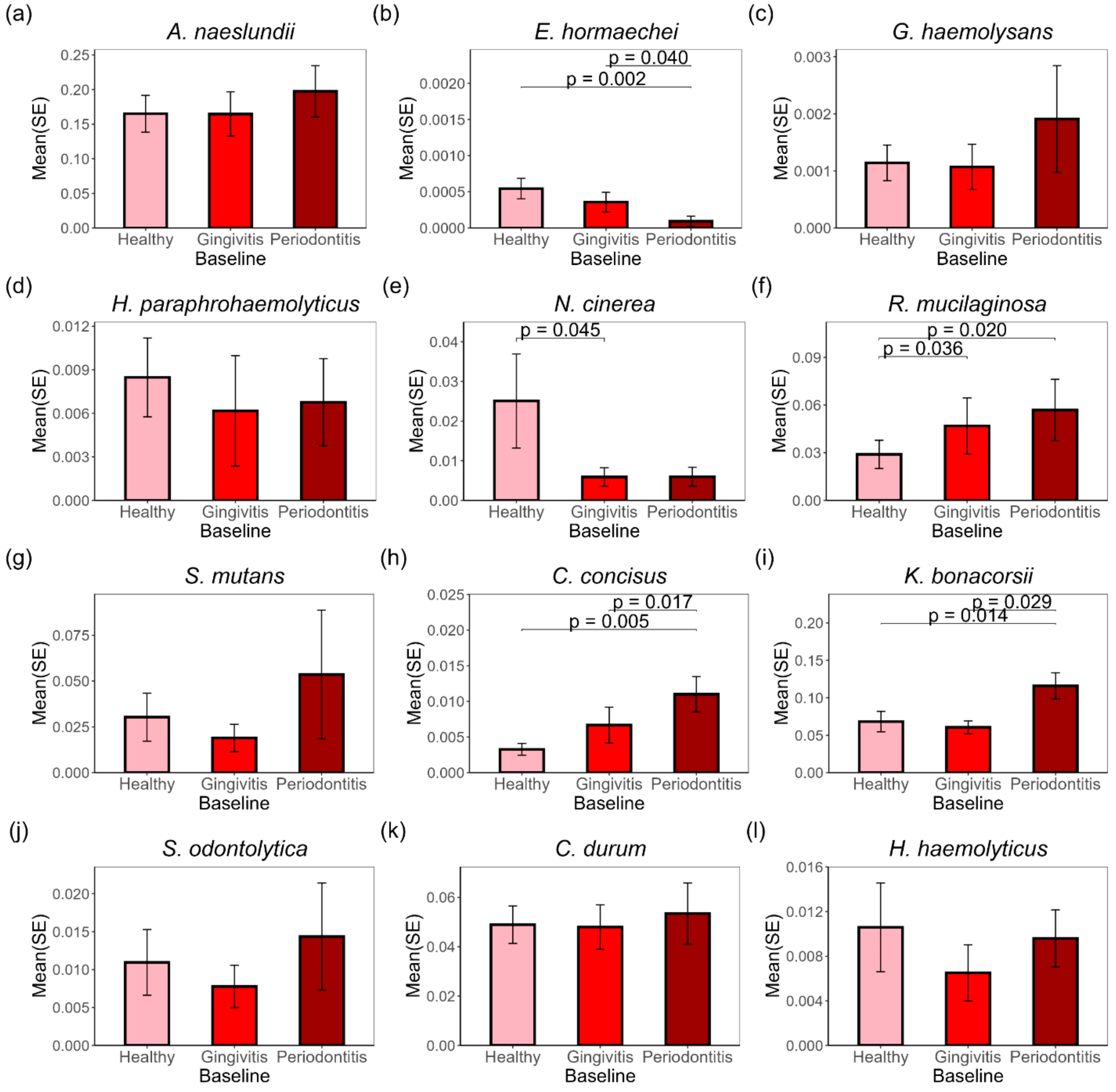

Figure S6. Correlation between the core bacteria in the red and pink constellations and the non-correlated bacteria in subgingival plaques at baseline with a p value of 0.05.

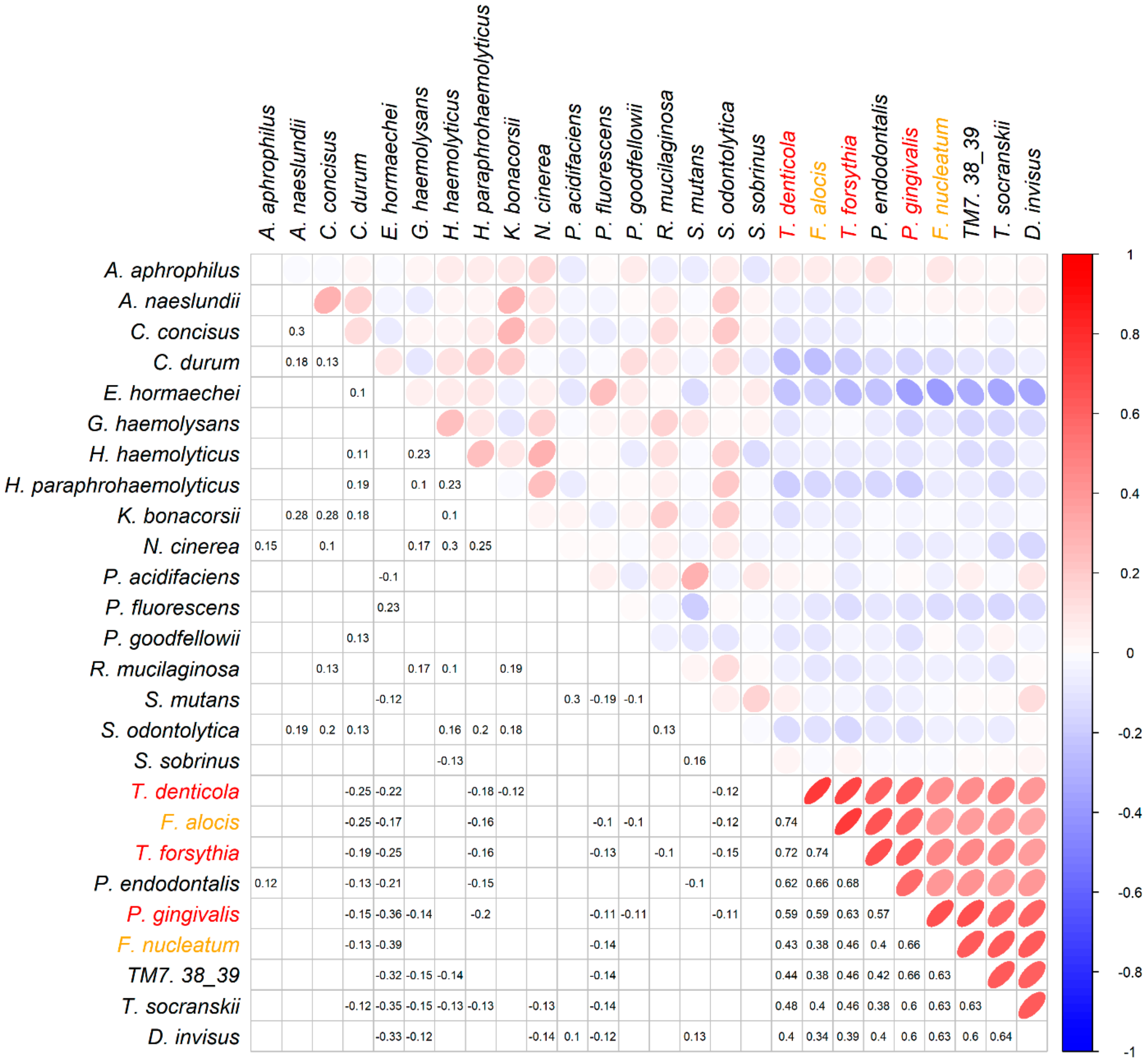

Blue means positive correlation while red is negative correlation.

Figure S7. Networks among the core bacteria in the red and pink constellations and the non-correlated bacteria in subgingival plaques at baseline with a p value of 0.05.

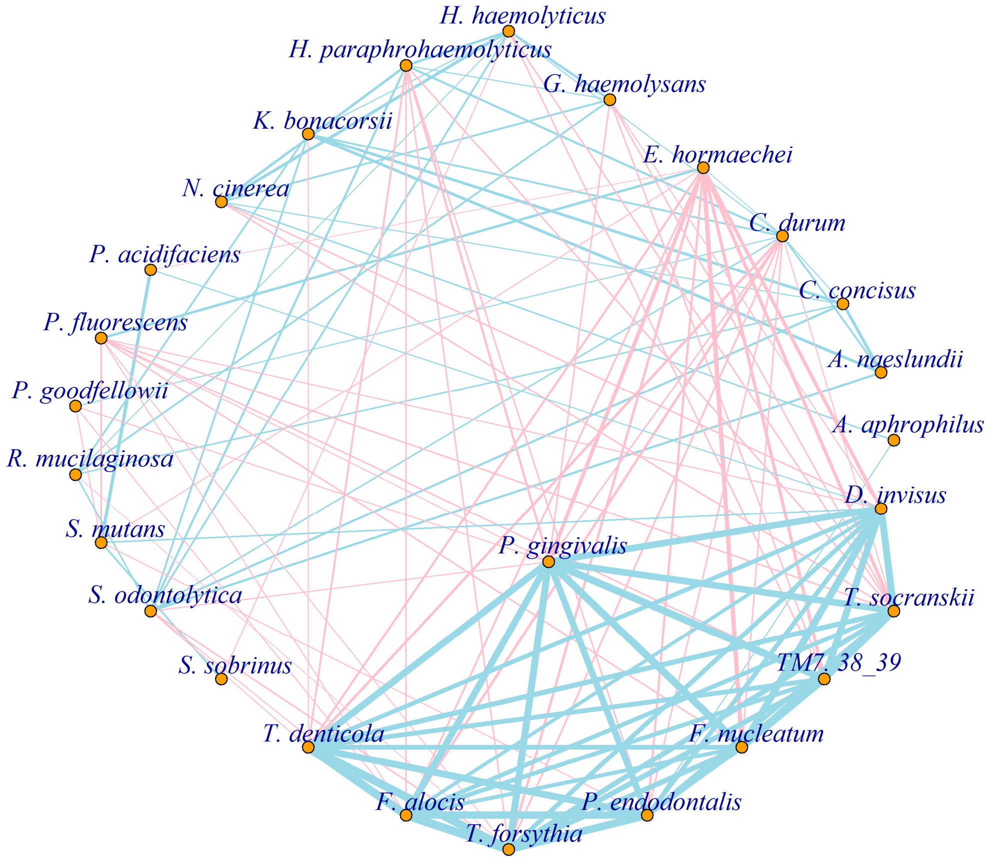

Blue indicates a positive correlation, while red signifies a negative correlation. The thickness of the lines corresponds to the magnitude of the correlation coefficients.

Figure S8. Comparison of bacterial abundance at genera levels between responders and non-responders at baseline and 24 months in subgingival plaques: Genera marked in blue indicate a significant reduction in abundance at 24 months (p < 0.01), while genera in green show a p-value of < 0.05 in the comparison of 24 months: Responders vs. Non-responders. For the remaining three columns (Responder: Month 24 vs. Baseline; Non-responder: Month 24 vs. Baseline; Baseline: Responder vs. Non-responder), only means and standard errors are presented.

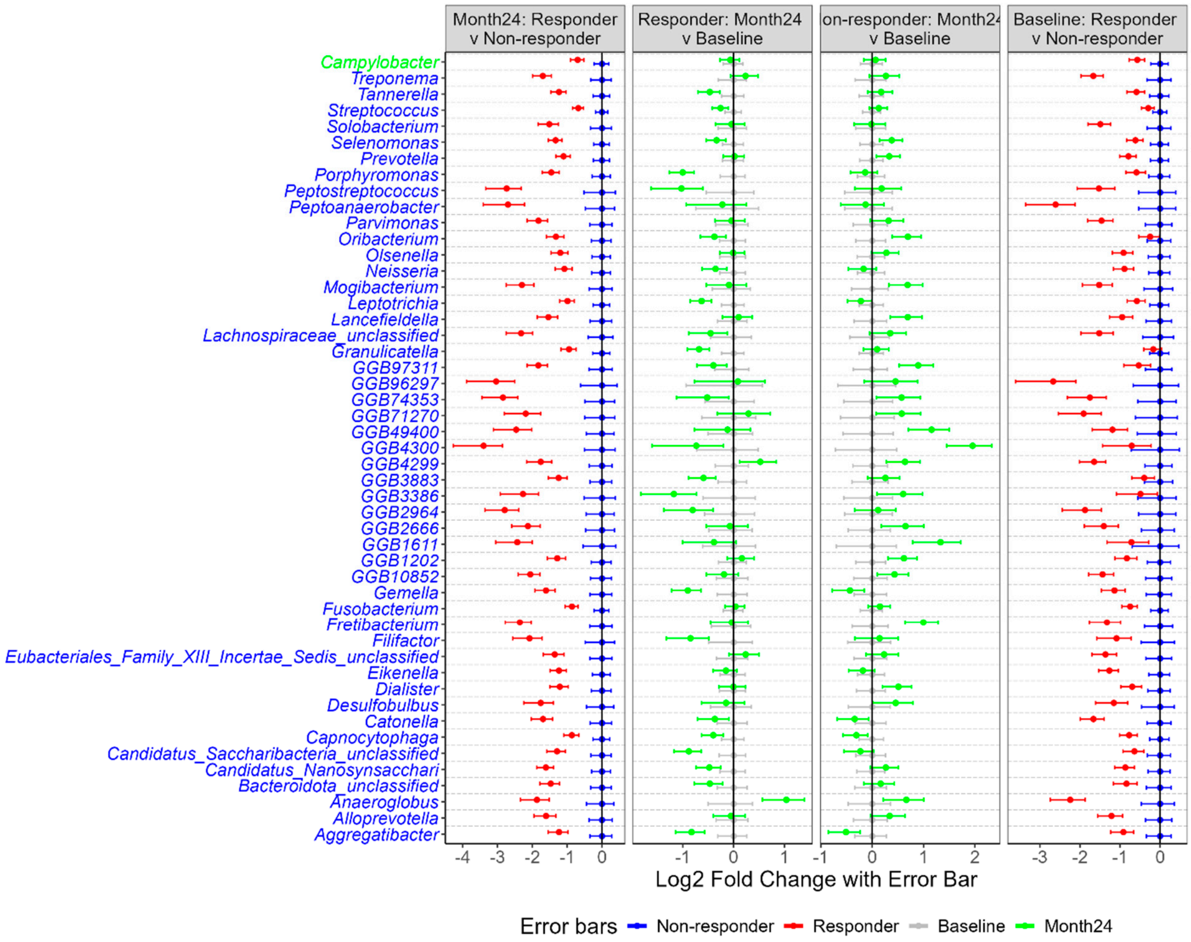

Supplement: Supplementary file 1 [file ijms-26-10823-s001.zip › Supplement Figures.pdf]
